# Multi-dimensional evidence establishing the causal association between metabolic syndrome and gout and the molecular mechanisms of comorbidity

**DOI:** 10.3389/fimmu.2026.1769138

**Published:** 2026-02-18

**Authors:** Jianbin Li, Jiamin Zhang, Suiran Li, Xiaoge Yao, Renhe Li, Wei Liu

**Affiliations:** 1 Department of Rheumatism and Immunity, First Teaching Hospital of Tianjin University of Traditional Chinese Medicine, Tianjin, China; 2 National Clinical Research Center for Chinese Medicine, Tianjin, China

**Keywords:** gout, metabolic syndrome, Mendelian randomization, transcriptomics, causal inference, risk prediction

## Abstract

**Objective:**

To systematically evaluate the causal association between metabolic syndrome (MetS) and its components with gout through integrated multi-dimensional methods, and reveal the genetic basis and transcriptomic characteristics of comorbidity.

**Methods:**

A three-phase research design was employed: (1) Real-world clinical cohort (n=8,853) was analyzed using propensity score matching (PSM), restricted cubic spline (RCS), and latent class trajectory modeling; (2) Two-sample Mendelian randomization (MR) and linkage disequilibrium score regression (LDSC) were applied for causal inference and genetic correlation assessment; (3) Transcriptomic data (GSE160170, GSE98895) were integrated for molecular mechanism analysis, with single-cell RNA sequencing data (GSE217561) used for hub gene cell-type specificity validation.

**Results:**

After PSM, MetS remained an independent risk factor for gout (OR = 1.456, 95%CI: 1.212-1.750, P<0.001), with hypertension (OR = 2.984) and hyperlipidemia (OR = 2.719) showing strongest associations. RCS analysis revealed significant non-linear relationships between metabolic indicators and gout risk. Trajectory analysis identified three triglyceride dynamic patterns, with the progressive elevation type showing significantly increased gout risk (HR = 1.92, P<0.001). MR analysis confirmed causal associations for MetS (OR = 1.171, P<0.001), hypertension (OR = 5.426, P = 0.002), triglycerides (OR = 1.325, P<0.001), and waist circumference (OR = 1.523, P<0.001), while HDL-C showed protective effect (OR = 0.887, P = 0.049); fasting blood glucose showed no significant causal association. LDSC revealed significant genetic correlation (rg=0.321, P = 4.24×10-15). Gene-level MR identified common risk genes including SNX11 and PGAP3, enriched in ABC transporters and immune regulatory pathways. Transcriptomic analysis identified core hub genes including JUN and FOS, enriched in Th17 cell differentiation and Toll-like receptor signaling pathways. Single-cell validation confirmed hub genes exhibited highest expression in monocytes and dendritic cells, with JUN, FOS, and IFNGR1 significantly upregulated in gout patients (P<0.0001), while TAP2 showed no expression change, supporting its pathogenic role through functional defects rather than transcriptional alterations.

**Conclusion:**

This study systematically established the causal association between MetS and gout through multi-dimensional evidence chains, revealing the molecular mechanism of comorbidity centered on antigen presentation-immune response and proposing a TAP2-UPR-Th17 pathological axis. These findings provide evidence-based support for early risk stratification and precision prevention of gout based on metabolic phenotypes.

## 1 Introduction

Gout is a metabolic inflammatory disease caused by monosodium urate (MSU) crystal deposition in joints and surrounding tissues, with clinical features including hyperuricemia, recurrent acute arthritis, tophi formation, and chronic joint damage. According to the Global Burden of Disease Study (GBD) 2021 data, the global prevalence of gout has reached 55.8 million, with age-standardized prevalence increasing by 22.5% since 1990, and projected to exceed 95.8 million by 2050 (1). This sustained upward trend makes gout a global public health concern requiring urgent attention.

Metabolic syndrome (MetS) is a cluster of metabolic abnormalities characterized by central obesity, insulin resistance, hypertension, dyslipidemia, and glucose metabolism disorders. Epidemiological data indicate that the global prevalence of MetS ranges from 11.9% to 49% and continues to rise (2, 3). Notably, numerous clinical studies have revealed a high degree of comorbidity between gout and MetS. A multicenter study by Jung et al. (4) showed that the prevalence of MetS in gout patients was as high as 43.6%, significantly higher than the 5.2% in the general population. Yoo et al. (5) further confirmed that gout patients had nearly three times higher rates of insulin resistance compared to healthy controls.

Although the epidemiological association between the two conditions has been widely reported, the directionality of causality and its biological mechanisms remain controversial. Traditional views suggest that MetS promotes gout development through multiple mechanisms: inflammatory factors produced by visceral adipose tissue can enhance hepatic uric acid synthesis (6); insulin resistance can increase uric acid reabsorption by upregulating renal tubular URAT1 expression (7); and hypertension affects uric acid excretion through renal microvascular damage (8). However, emerging evidence suggests that hyperuricemia itself may also serve as a precursor factor for MetS, participating in MetS development through promoting endothelial dysfunction, activating the renin-angiotensin system, and increasing oxidative stress (9). Therefore, clarifying the causal relationship between them is of important scientific significance for understanding disease mechanisms and developing targeted intervention strategies.

Previous studies investigating the association between MetS and gout have several methodological limitations. First, most observational studies have limited sample sizes from single sources, making it difficult to effectively control confounding factors, weakening the reliability of conclusions (10). Second, traditional analyses are mostly based on linear assumptions, ignoring possible non-linear dose-response relationships and threshold effects, which are crucial for clinically determining intervention cutoff points. Third, few studies have systematically explored the modifying effects of population heterogeneity (such as age and gender) on association strength. Fourth, due to the inherent defects of observational study design, it is difficult to exclude the influence of reverse causation and unmeasured confounding, making it impossible to establish definite causal relationships (11). Finally, previous studies have mostly focused on epidemiological association descriptions, lacking in-depth exploration of the molecular mechanisms underlying the comorbidity of these two diseases.

Mendelian randomization (MR), as a causal inference method using genetic variants as instrumental variables, can effectively overcome the limitations of traditional observational studies. Since genetic variants are randomly allocated during gamete formation and are already determined at birth, MR analysis can maximize the avoidance of reverse causation and confounding bias, providing more reliable evidence for causal inference (12, 13). Additionally, integrating transcriptomic data for multi-omics analysis can validate genetic findings and reveal potential molecular mechanisms at the gene expression level, providing a more comprehensive biological explanation for understanding disease comorbidity.

Based on the above considerations, this study establishes a stepwise causal evidence framework of “Observation-Validation-Mechanism,” rather than a simple superposition of multiple methods. First, we utilized a large real-world clinical cohort (n=8,853) to capture the macroscopic associations and non-linear characteristics between MetS and gout. Subsequently, addressing the limitations of observational studies regarding confounding, we applied Mendelian Randomization (MR) to confirm the causal nature of these associations and filter out spurious links caused by environmental confounders (distinguishing the true from the false). Finally, we delved into the transcriptomic level to decipher the molecular landscape behind the causality, verifying how genetic signals translate into specific immuno-metabolic disorders. This progressive strategy aims to build a coherent and complete causal evidence chain, providing a solid evidence-based foundation for early risk stratification, precision prevention, and targeted treatment of gout.

## 2 Methods

### 2.1 Study design and ethics statement

This study employed a multi-phase, multi-method integrated research design, including three mutually validating analytical modules: (1) real-world clinical cohort study; (2) Mendelian randomization genetic analysis; (3) Transcriptomics and single-cell bioinformatics analysis. Real-world study data were obtained from the electronic medical record system of the First Teaching Hospital of Tianjin University of Traditional Chinese Medicine, covering patients with complete medical records who received treatment between 2014 and 2024. This study was approved by the hospital ethics committee, and informed consent was waived given the retrospective nature of the study. Genetic data used in MR analysis were all derived from published genome-wide association study (GWAS) summary statistics, and the relevant original studies had all received ethical approval.

### 2.2 Real-world clinical cohort study

#### 2.2.1 Study population and inclusion/exclusion criteria

Inclusion criteria: (1) Gout group: Meeting the American College of Rheumatology (ACR)/European League Against Rheumatism (EULAR) 2015 gout classification criteria (14), with complete clinical and laboratory records; (2) Control group: Non-gout patients who visited our hospital during the same period, with no history of gout. Exclusion criteria included: (1) Unclear gout diagnosis or lack of supporting evidence; (2) Missing key clinical data required for MetS assessment; (3) Comorbid other inflammatory joint diseases (such as rheumatoid arthritis, ankylosing spondylitis); (4) Severe hepatic or renal insufficiency (end-stage renal disease, cirrhosis); (5) Active malignancy; (6) Age <18 years. A total of 8,853 subjects were finally included, with 4,114 in the gout group and 4,739 in the non-gout group.

#### 2.2.2 Variable definitions and data collection

The main exposure variable was MetS, with diagnostic criteria according to the International Diabetes Federation (IDF) 2005 global consensus (15). The primary outcome variable was gout occurrence, confirmed through elevated serum uric acid, typical clinical symptoms, and/or joint fluid examination and imaging studies. Collected data included: demographic characteristics (age, gender), comorbidity status (hypertension, diabetes, hyperlipidemia), laboratory indicators (fasting blood glucose, total cholesterol, triglycerides, HDL-C, LDL-C, serum uric acid, liver and kidney function indicators), and medication status (antihypertensive, hypoglycemic, lipid-lowering drugs).

#### 2.2.3 Propensity score matching

To control baseline confounding factors, propensity score matching (PSM) was used. A propensity score model was constructed based on clinically relevant variables including gender, age, and MetS status, using the nearest neighbor matching algorithm (caliper=0.2) for 1:1 matching without replacement. Matching quality was evaluated by standardized mean difference (SMD), with SMD<0.1 considered to indicate good balance (16). A total of 3,010 matched samples were finally obtained (1,505 in each group).

#### 2.2.4 Non-linear relationships and subgroup analysis

Restricted cubic spline (RCS) models were used to explore non-linear dose-response relationships between metabolic indicators and gout risk, with knots placed at the 5th, 35th, 65th, and 95th percentiles. Non-linearity testing was based on the Wald test, with P<0.05 indicating significant non-linear relationships. Subgroup analyses were performed by age (<60 years, ≥60 years) and gender to assess population heterogeneity in association strength, with interaction effects assessed by likelihood ratio tests.

#### 2.2.5 Latent class trajectory modeling and dynamic risk assessment

To identify heterogeneous longitudinal trajectory patterns of triglycerides (TG) over time, we employed latent class mixed models using the lcmm package in R. Given the skewed distribution of TG data, logarithmic transformation was performed prior to modeling. Within a mixed-effects framework, log-transformed TG was modeled as the dependent variable with follow-up time as the independent variable. To capture the non-linear dynamic characteristics of metabolic indicators, we included quadratic terms of time in both fixed and random effects. We fitted models ranging from 1 to 4 latent classes, and the optimal number of classes was determined based on a comprehensive set of criteria: minimization of the Bayesian Information Criterion (BIC), an average posterior probability (AvePP) greater than 0.7 for each class to ensure high classification confidence, and a minimum class proportion greater than 5% to exclude spurious small clusters. The identified trajectory classes were then extracted as categorical variables and entered into a multivariable Cox proportional hazards model to evaluate the independent predictive value of different TG dynamic patterns on gout risk, adjusting for baseline covariates including age, sex, hypertension, diabetes, and hyperlipidemia, with results presented as Hazard Ratios (HR) and 95% Confidence Intervals (CI).

### 2.3 Mendelian randomization analysis

#### 2.3.1 Data sources

GWAS data for exposure variables (MetS and its components) were derived from the IEU Open GWAS project (https://gwas.mrcieu.ac.uk) and GWAS Catalog (https://www.ebi.ac.uk/gwas). Specifically included: MetS (UK Biobank), waist circumference (UK Biobank), hypertension (UK Biobank), fasting blood glucose (MAGIC Consortium), triglycerides (Global Lipids Genetics Consortium), and HDL-C (Global Lipids Genetics Consortium). GWAS data for the outcome variable gout were derived from FinnGen database Release 11 (https://risteys.finregistry.fi) All data were based on European ancestry populations to avoid population stratification bias. See **Supplementary Table 1** for details.

#### 2.3.2 Instrumental variable selection

MR analysis requires satisfying three core assumptions: (1) Relevance assumption: genetic variants are strongly associated with exposure variables; (2) Independence assumption: genetic variants are not associated with confounding factors; (3) Exclusion restriction assumption: genetic variants affect outcomes only through exposure (17). Accordingly, instrumental variable selection criteria were as follows: (1) Genome-wide significance threshold P<5×10^-8^; (2) Linkage disequilibrium coefficient r²<0.001, clumping window 10,000 kb; (3) F-statistic >10, ensuring instrumental variable strength; (4) Radial MR to remove outliers. Proxy SNPs were not included to avoid potential bias.

#### 2.3.3 Statistical methods and sensitivity analysis

Causal effect estimation used inverse variance weighted (IVW) method, MR-Egger regression, and weighted median method (18). Among these, the IVW method has the highest statistical power under the assumption of no horizontal pleiotropy and was used as the primary analysis method. Sensitivity analyses included: (1) Cochran’s Q statistic to assess heterogeneity (P>0.05 indicating no significant heterogeneity); (2) MR-Egger intercept test and MR-PRESSO global test to assess horizontal pleiotropy; (3) Leave-one-out analysis to assess the influence of individual SNPs.

#### 2.3.4 Genetic correlation analysis

Linkage disequilibrium score regression (LDSC) analysis was used to quantify genome-wide genetic correlation between MetS and gout (19). GWAS summary statistics were harmonized with the HapMap3 reference panel to estimate single-trait heritability (h²) and cross-trait genetic correlation coefficient (rg). To correct for ascertainment bias in case-control designs, observed heritability was converted to liability scale, with MetS population prevalence set at 25% and gout at 2%.

#### 2.3.5 Gene-level Mendelian randomization

To identify “common pathogenic genes” that simultaneously affect MetS and gout, a gene-level MR strategy was employed. Genome-wide gene-level MR screening was performed separately for MetS and gout, with selection criteria of IVW P<0.05 and consistent effect direction across all sensitivity analyses. Venn diagram intersection identified common risk genes (both OR>1) and common protective genes (both OR<1).

### 2.4 Transcriptomic bioinformatics analysis

#### 2.4.1 Data acquisition and preprocessing

Transcriptomic data based on peripheral blood mononuclear cells (PBMC) were obtained from the Gene Expression Omnibus (GEO) database to minimize background noise from tissue heterogeneity. Specifically included: gout dataset GSE160170 (6 patients *vs* 6 healthy controls) and MetS dataset GSE98895 (20 patients *vs* 20 healthy controls). Raw data underwent background correction and quantile normalization.

#### 2.4.2 Differential expression analysis

Differential expression analysis was performed using the R package limma Significantly differentially expressed genes (DEGs) were defined as adjusted P-value (using Benjamini-Hochberg correction) <0.05 and |log_2_FC|>0.585 (i.e., fold change >1.5). Venn diagrams were used to extract common differentially expressed genes with consistent expression trends in both diseases as the basis for subsequent comorbidity mechanism analysis.

#### 2.4.3 Protein interaction network construction and core gene screening

Common differentially expressed genes were imported into the STRING database (v11.5) to construct protein-protein interaction (PPI) networks, with minimum interaction confidence set at 0.4 and isolated nodes removed. Cytoscape software was used for visualization, and the cytoHubba plugin’s 10 topological analysis algorithms (MCC, MNC, Degree, EPC, BottleNeck, EcCentricity, Closeness, Radiality, Betweenness, Stress) were used for comprehensive scoring. The UpSetR package was used to intersect the top 20 genes identified by each algorithm, with genes identified by all algorithms defined as core hub genes.

#### 2.4.4 Functional enrichment analysis

The clusterProfiler package was used to perform Gene Ontology (GO) and Kyoto Encyclopedia of Genes and Genomes (KEGG) pathway enrichment analysis on common differentially expressed genes and hub genes. The significance threshold was set at P<0.05. GO analysis covered three categories: biological process (BP), cellular component (CC), and molecular function (MF).

#### 2.4.5 Transcription factor regulatory network construction

Key transcription factors regulating hub genes were predicted based on the TRRUST database (v2.0) to construct a “transcription factor-hub gene” multi-dimensional regulatory network. The GeneMANIA platform was used to construct co-expression, physical interaction, and co-localization networks of hub genes, validating their internal connectivity as functional modules.

### 2.5 Statistical analysis

**Figure 1:**
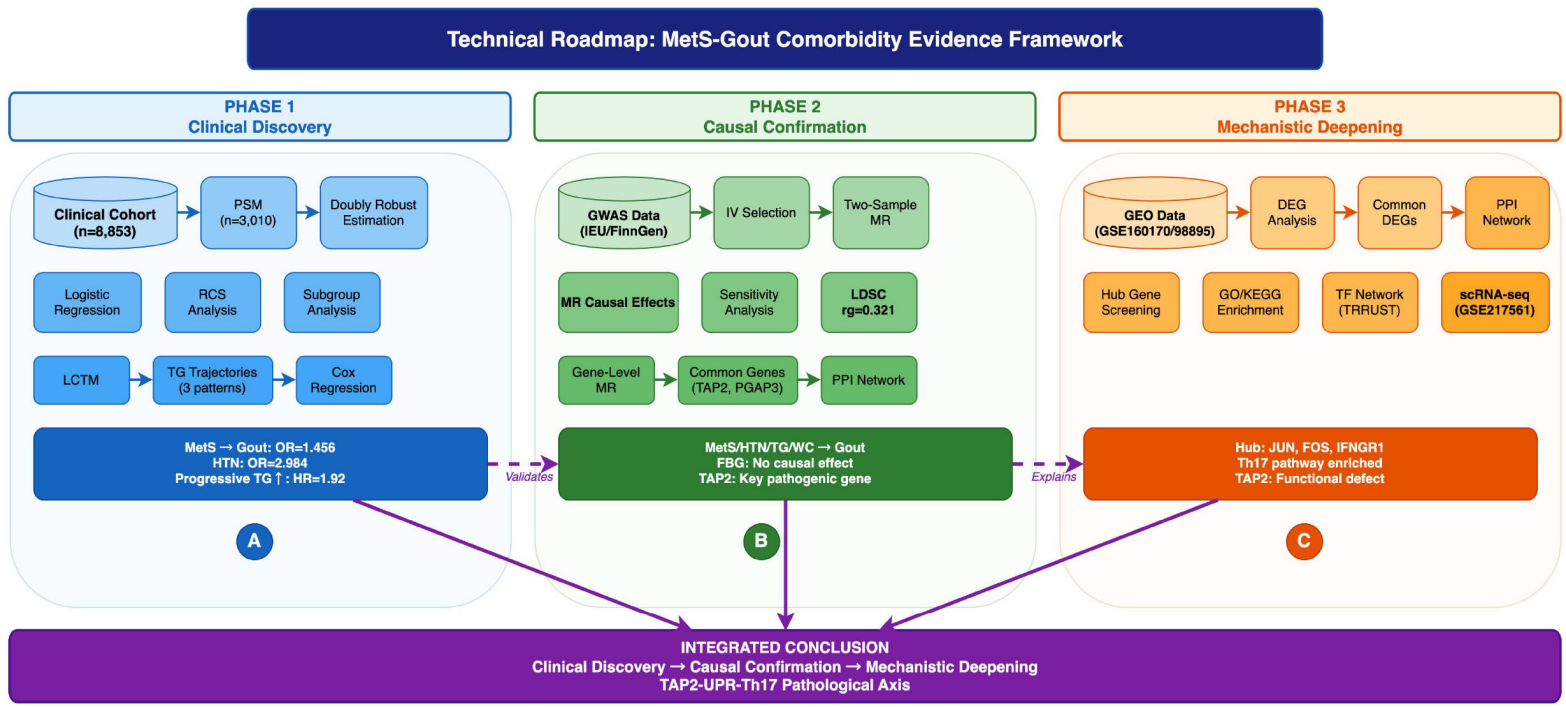
Research technical roadmap. This study employs a three-stage design to systematically evaluate the comorbidity mechanisms between metabolic syndrome and gout: **(A)** Phase I involves the identification of high-risk metabolic subtypes within a large real-world clinical cohort (n=8,853) using Propensity Score Matching (PSM) and Latent Class Trajectory Modeling (LCTM); **(B)** Phase II applies Two-Sample Mendelian Randomization (MR) and Linkage Disequilibrium Score Regression (LDSC) to confirm causal associations and eliminate environmental confounders; and **(C)** Phase III integrates transcriptomic data analysis with single-cell validation.

All statistical analyses were performed using R software (v4.2.0). Continuous variables were expressed as mean ± standard deviation or median (interquartile range), with between-group comparisons using t-test or Mann-Whitney U test; categorical variables were expressed as frequency (percentage), with between-group comparisons using χ² test. Multivariable analysis used logistic regression or Cox regression models. Given the residual imbalance in age after propensity score matching (SMD > 0.1), we employed a “doubly robust estimation” strategy by including age as a mandatory covariate in the subsequent multivariable regression models. This approach ensures that any residual confounding bias not fully eliminated by the matching process is statistically adjusted for, thereby providing unbiased and robust effect estimates. Two-sided P<0.05 was considered statistically significant. MR analysis used TwoSampleMR, MR-PRESSO, and ldscr packages; transcriptomic analysis used limma, clusterProfiler, and Cytoscape (v3.9.1).The overall research roadmap is illustrated in **Figure 1**.

## 3 Results

### 3.1 Propensity score matching results

**Figure 2:**
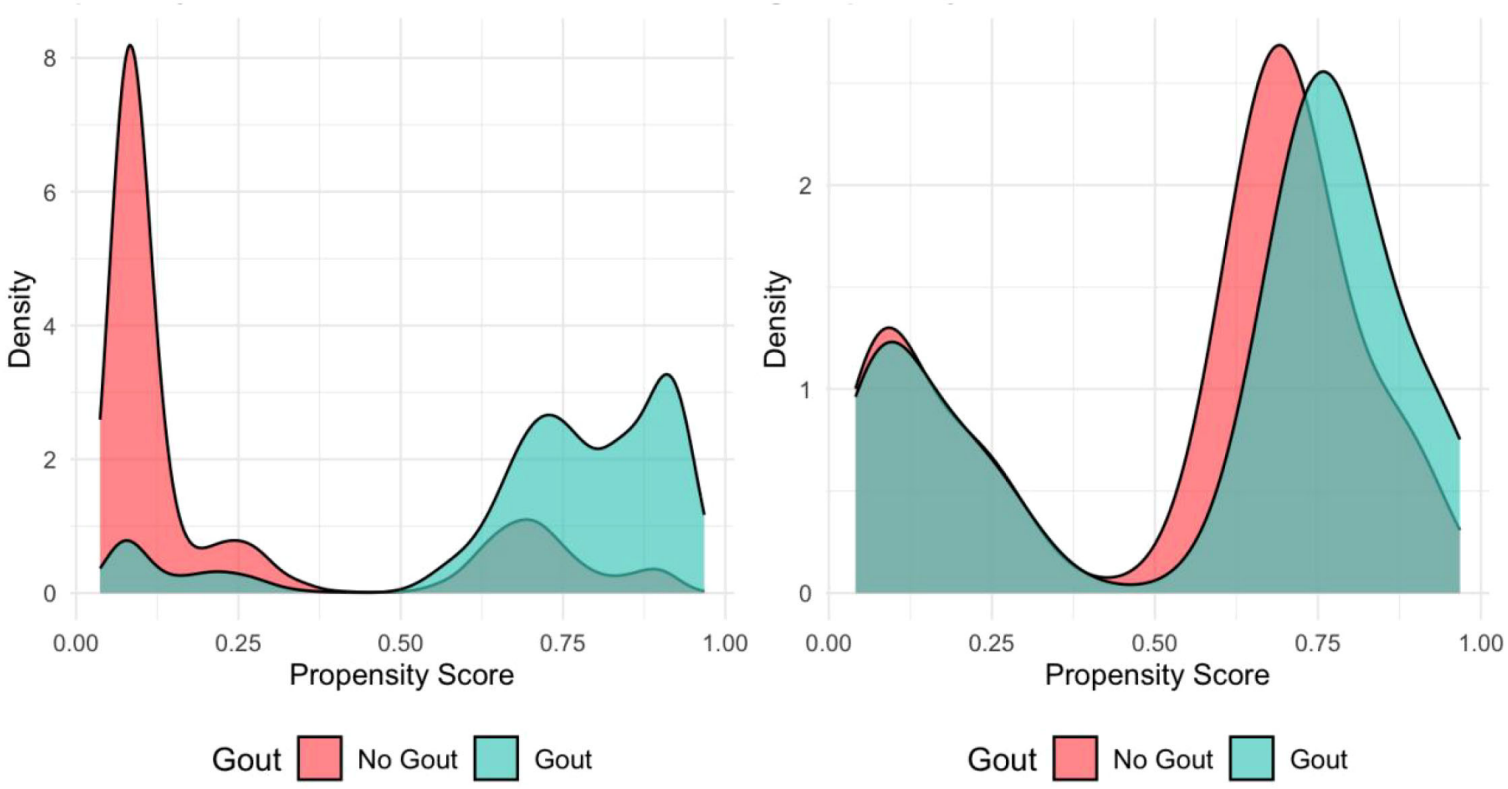
Propensity score distribution comparison before and after matching between gout and non-gout groups.

To effectively control baseline confounding factors, this study employed propensity score matching. Before matching, the propensity score distributions of the gout and non-gout groups showed significant differences; after 1:1 matching, the score distribution curves of the two groups highly overlapped, indicating that baseline imbalance had been effectively corrected (**Figure 2**). A total of 3,010 matched samples (1,505 in each group) were finally included for subsequent analysis. Matching quality assessment showed that gender (standardized mean difference [SMD]=0.0083) and metabolic syndrome status (SMD = 0.1189) had achieved good balance between groups. However, the age variable still showed some residual imbalance after matching (SMD = 0.4880), so it was included as a covariate in subsequent multivariable regression analyses.

### 3.2 Baseline characteristics of study population

This real-world study included a total of 8,852 subjects. Before PSM, the gout group (n=4,114) and non-gout group (n=4,738) showed statistically significant differences in baseline characteristics including age, gender, comorbidities, and multiple metabolic indicators (**Table 1**). After 1:1 PSM, 3,010 paired samples were finally obtained for analysis. After matching, good balance was achieved between groups for indicators such as gender, diabetes prevalence, and total cholesterol levels. However, it is noteworthy that even after strict matching, gout patients still showed significantly higher metabolic syndrome prevalence, hypertension and hyperlipidemia rates, along with higher fasting blood glucose and triglyceride levels, and lower HDL-C levels (all P<0.05). Detailed baseline characteristics comparison after matching is shown in **Table 2**.

**Table 1:** Baseline characteristics comparison between gout and non-gout groups before propensity score matching.

Characteristic	Total	Gout	Non-Gout	P
N	8852	4114	4738	–
Male, n(%)	4652	3632	1020	<0.001
Age (years), mean ± SD	60.03 ± 60.59	56.92 ± 57.82	62.53 ± 63.17	<0.001
Metabolic syndrome, n(%)	2123	1414	709	<0.001
SBP (mmHg), mean ± SD	–	139.80 ± 20.12	–	–
DBP (mmHg), mean ± SD	–	87.94 ± 17.96	–	–
Hypertension, n(%)	4186	2503	1683	<0.001
Diabetes, n(%)	1396	794	575	<0.001
Hyperlipidemia, n(%)	1169	845	324	<0.001
FBG (mmol/L), mean ± SD	5.74 ± 2.01	6.14 ± 2.19	5.39 ± 1.75	<0.001
TC (mmol/L), mean ± SD	4.54 ± 1.25	4.41 ± 1.22	4.65 ± 1.27	<0.001
TG (mmol/L), mean ± SD	1.73 ± 1.79	2.18 ± 1.89	1.34 ± 1.61	<0.001
HDL-C (mmol/L), mean ± SD	1.06 ± 0.32	0.95 ± 0.25	1.15 ± 0.33	<0.001
LDL-C (mmol/L), mean ± SD	2.74 ± 0.92	2.63 ± 0.92	2.85 ± 0.91	<0.001
ALT (U/L), mean ± SD	23.39 ± 24.86	29.49 ± 30.00	18.02 ± 17.57	<0.001
AST (U/L), mean ± SD	21.14 ± 20.36	23.19 ± 24.01	19.35 ± 16.29	<0.001
UA (μmol/L), mean ± SD	–	462.77 ± 134.23	–	–
Cr (μmol/L), mean ± SD	–	108.19 ± 102.49	–	–
BUN (mmol/L), mean ± SD	–	6.79 ± 4.67	–	–
CRP (mg/L), mean ± SD	–	30.07 ± 47.13	–	–
ESR (mm/h), mean ± SD	–	35.78 ± 30.88	–	–
Gout duration (years), mean ± SD	–	5.39 ± 3.01	–	–
Medication use				
Lipid-lowering drugs, n(%)	1100	576	524	<0.001
Hypoglycemic drugs, n(%)	1418	742	676	<0.001
Antihypertensive drugs, n(%)	2177	1392	785	<0.001

SBP, systolic blood pressure; DBP, diastolic blood pressure; FBG, fasting blood glucose; TC, total cholesterol; TG, triglycerides; HDL-C, high-density lipoprotein cholesterol; LDL-C, low-density lipoprotein cholesterol; ALT, alanine aminotransferase; AST, aspartate aminotransferase; UA, uric acid; Cr, creatinine; BUN, blood urea nitrogen; CRP, C-reactive protein; ESR, erythrocyte sedimentation rate; SD, standard deviation.

**Table 2:** Baseline characteristics comparison between gout and non-gout groups after propensity score matching.

Characteristic	Total	Gout	Non-Gout	P
N	3010	1505	1505	–
Male, n(%)	2044	1024	1020	0.876
Age (years), mean ± SD	–	–	–	<0.001
Metabolic syndrome, n(%)	687	386	301	<0.001
SBP (mmHg), mean ± SD	–	138.41 ± 20.29	–	–
DBP (mmHg), mean ± SD	–	87.01 ± 12.82	–	–
Hypertension, n(%)	1407	848	559	<0.001
Diabetes, n(%)	490	263	227	0.076
Hyperlipidemia, n(%)	406	300	106	<0.001
FBG (mmol/L), mean ± SD	5.80 ± 2.08	5.99 ± 2.06	5.62 ± 2.09	<0.001
TC (mmol/L), mean ± SD	4.49 ± 1.22	4.53 ± 1.22	4.47 ± 1.22	0.195
TG (mmol/L), mean ± SD	1.74 ± 2.20	2.14 ± 1.70	1.35 ± 2.53	<0.001
HDL-C (mmol/L), mean ± SD	1.04 ± 0.31	1.00 ± 0.26	1.08 ± 0.34	<0.001
LDL-C (mmol/L), mean ± SD	2.73 ± 0.92	2.69 ± 0.94	2.78 ± 0.91	0.007
ALT (U/L), mean ± SD	23.73 ± 22.28	27.86 ± 24.41	19.68 ± 19.13	<0.001
AST (U/L), mean ± SD	20.83 ± 15.05	21.97 ± 14.18	19.70 ± 15.79	<0.001
UA (μmol/L), mean ± SD	–	463.84 ± 138.98	–	–
Cr (μmol/L), mean ± SD	–	109.61 ± 114.88	–	–
BUN (mmol/L), mean ± SD	–	6.98 ± 5.10	–	–
CRP (mg/L), mean ± SD	–	26.59 ± 43.55	–	–
ESR (mm/h), mean ± SD	–	35.57 ± 31.05	–	–
Gout duration (years), mean ± SD	–	3.98 ± 2.82	–	–
Medication use				
Lipid-lowering drugs, n(%)	521	241	280	0.060
Hypoglycemic drugs, n(%)	492	249	243	0.767
Antihypertensive drugs, n(%)	959	573	386	<0.001

### 3.3 Subgroup analysis of associations between metabolic syndrome and its components with gout

To explore population heterogeneity in depth, this study performed stratified subgroup analysis of the associations between metabolic syndrome and its components with gout (**Table 3**). The study found that age was an important effect modifier: hyperlipidemia and total cholesterol had more significant effects on gout risk in younger populations (<60 years) (interaction P values of 0.007 and <0.001, respectively), while LDL-C showed a protective effect only in older populations (interaction P = 0.004).

**Table 3:** Subgroup analysis of associations between metabolic syndrome and its components with gout by age and gender.

Variable	Age<60 OR (95%CI)	Age≥60 OR (95%CI)	P-int age	Male OR (95%CI)	Female OR (95%CI)	P-int gender
MetS	1.72(1.26,2.37)	1.47(1.18,1.83)	0.431	1.73(1.37,2.20)	1.07(0.82,1.39)	0.007
DM	1.75(1.20,2.61)	1.26(0.99,1.60)	0.151	1.06(0.82,1.37)	1.42(1.05,1.92)	0.145
HDL	0.40(0.26,0.61)	0.51(0.37,0.70)	0.375	0.50(0.36,0.68)	0.33(0.21,0.50)	0.121
HTN	2.97(2.29,3.89)	2.83(2.32,3.46)	0.772	2.12(1.78,2.53)	2.33(1.80,3.02)	0.546
FBG	1.20(1.11,1.31)	1.09(1.04,1.14)	0.041	1.11(1.06,1.16)	1.07(1.00,1.15)	0.417
LDL	1.02(0.89,1.17)	0.80(0.72,0.89)	0.004	0.92(0.83,1.01)	0.87(0.75,1.00)	0.539
TC	1.18(1.06,1.32)	0.95(0.88,1.02)	<0.001	1.11(1.02,1.20)	0.97(0.88,1.07)	0.046
Hyperlipidemia	4.27(2.91,6.45)	2.15(1.57,2.96)	0.007	4.74(3.54,6.43)	1.49(1.00,2.23)	<0.001

MetS, metabolic syndrome; DM, diabetes mellitus; HDL, high-density lipoprotein; HTN, hypertension; FBG, fasting blood glucose; LDL, low-density lipoprotein; TC, total cholesterol; OR, odds ratio; CI, confidence interval; P-int, P for interaction.

Gender differences were also significant. The association between metabolic syndrome and gout reached statistical significance only in males (OR = 1.73, 95%CI: 1.37-2.20), and interaction tests also confirmed this gender difference (interaction P = 0.007). Similarly, the effects of hyperlipidemia and total cholesterol on male gout risk were significantly higher than in females (interaction P<0.05). In contrast, hypertension was a stable risk factor across all age and gender subgroups, while low HDL-C showed protective effects in both sexes, with no significant interactions observed.

### 3.4 Multivariable regression analysis of gout-related factors

In the multivariable logistic regression analysis of real-world cohort data, this study systematically evaluated the independent associations of various metabolic factors with gout (**Table 4**). After adjusting for potential confounders including age and gender, metabolic syndrome itself remained an independent risk factor for gout (adjusted OR = 1.456, 95%CI: 1.212-1.750, P<0.001). Among metabolic syndrome components, hypertension (adjusted OR = 2.984, 95%CI: 2.542-3.502) and hyperlipidemia (adjusted OR = 2.719, 95%CI: 2.136-3.460) showed the strongest associations with gout risk. Additionally, elevated fasting blood glucose (adjusted OR = 1.108), elevated triglycerides (adjusted OR = 1.664), and abnormal HDL-C (adjusted OR = 0.445) and LDL-C (adjusted OR = 0.854) levels were all independently associated with gout risk (all P<0.01).

**Table 4:** Univariable and multivariable logistic regression analysis results for gout-related factors.

Characteristic	Univariable OR(95%CI)	P	Multivariable OR(95%CI)	P
Age	0.961(0.955-0.966)	<0.001	–	–
Gender	1.012(0.869-1.180)	0.876	–	–
Metabolic syndrome	1.380(1.163-1.638)	<0.001	1.456(1.212-1.750)	<0.001
Diabetes	1.192(0.982-1.447)	0.076	–	–
Hypertension	2.184(1.888-2.528)	<0.001	2.984(2.542-3.502)	<0.001
Hyperlipidemia	3.286(2.599-4.154)	<0.001	2.719(2.136-3.460)	<0.001
Fasting blood glucose	1.094(1.052-1.137)	<0.001	1.108(1.063-1.154)	<0.001
HDL-C	0.424(0.330-0.545)	<0.001	0.445(0.342-0.579)	<0.001
LDL-C	0.897(0.828-0.971)	0.007	0.854(0.785-0.928)	<0.001
Total cholesterol	1.041(0.980-1.105)	0.195	–	–
Triglycerides	1.802(1.650-1.968)	<0.001	1.664(1.522-1.820)	<0.001

### 3.5 Non-linear dose-response relationships between metabolic parameters and gout risk

**Figure 3:**
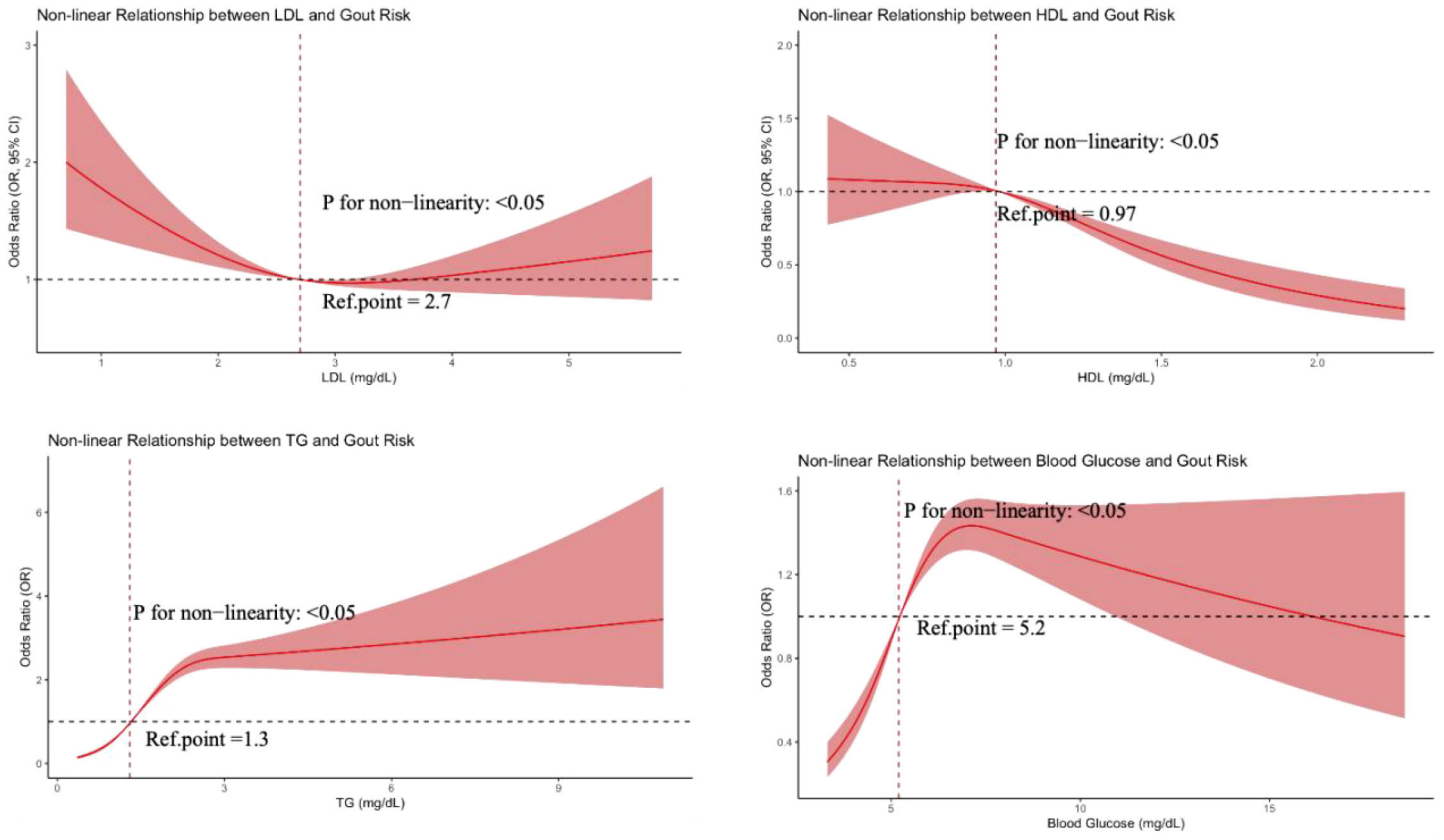
Non-linear dose-response relationships between metabolic parameters and gout risk.

This study systematically analyzed dose-response relationships between metabolic parameters and gout risk using restricted cubic spline (RCS) models. As shown in **Figure 3**, all metabolic parameters showed significant non-linear associations with gout risk (non-linearity test P<0.05). Specifically: HDL-C was negatively associated with gout risk, with risk decreasing as concentration increased using 0.97 mmol/L as reference; LDL-C showed a U-shaped relationship with gout risk, with lowest risk around 2.7 mmol/L; triglycerides were positively associated with gout risk, with risk increasing as concentration increased using 1.3 mmol/L as reference; fasting blood glucose showed an inverted J-shaped relationship with gout risk, with risk rising sharply in the hypoglycemic range and slowly declining in the hyperglycemic range using 5.2 mmol/L as reference. These results reveal clear threshold effects in the associations between metabolic parameters and gout risk, providing important guidance for precise clinical risk assessment and individualized prevention strategies.

### 3.6 Identification of dynamic triglyceride trajectories and their predictive value for gout risk

**Figure 4:**
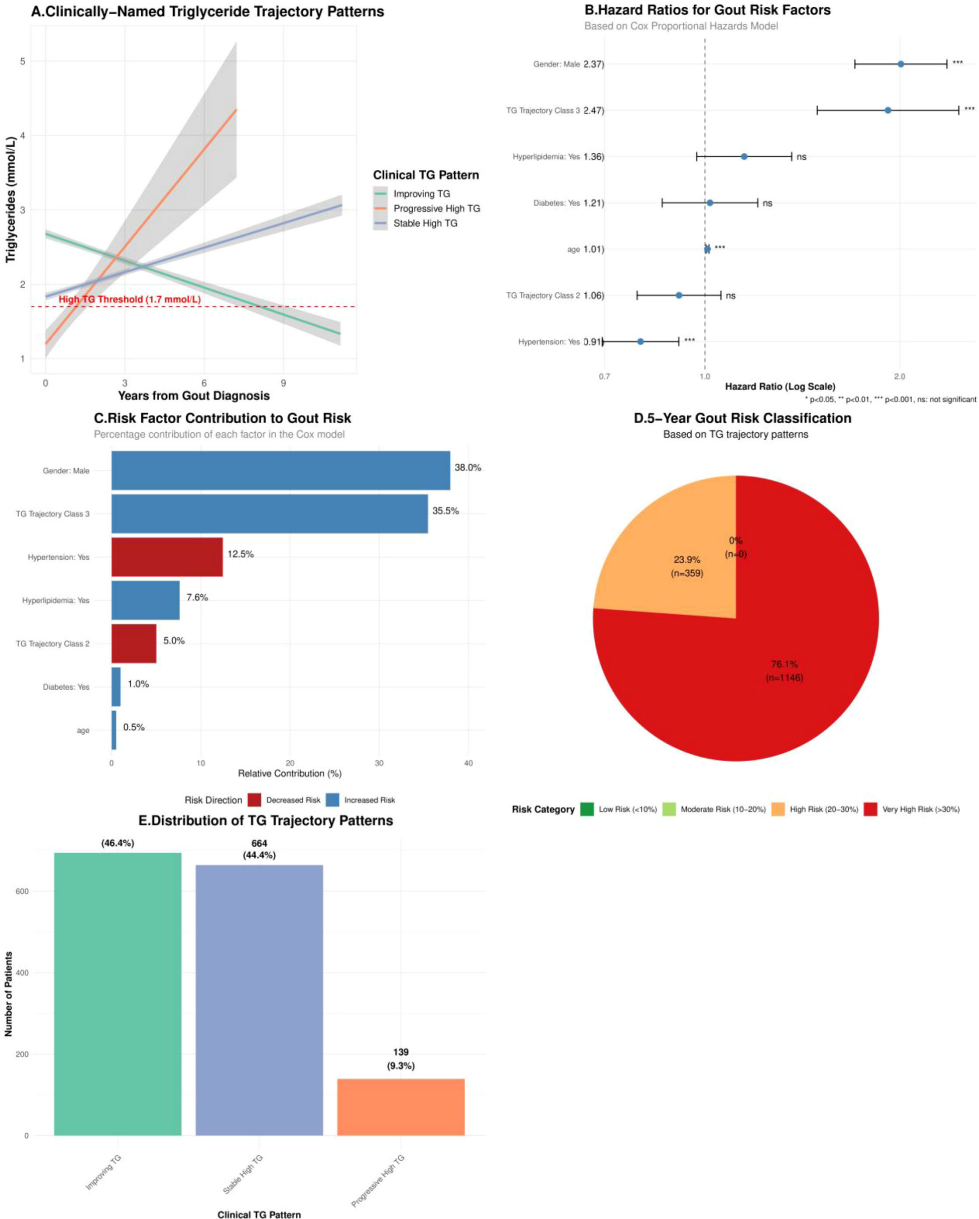
Comprehensive report of gout risk analysis and prediction based on dynamic triglyceride trajectories. **(A)** Three TG dynamic trajectory patterns identified by latent class mixed models, with the red dashed line representing the clinical threshold for hypertriglyceridemia (1.7 mmol/L). **(B)** Patient distribution and proportions of the three TG trajectory patterns in the study cohort. **(C)** Forest plot of multivariable Cox regression model showing hazard ratios (HR) and 95% confidence intervals for gout occurrence including TG trajectory and other variables. **(D)** Machine learning model variable importance analysis showing relative contributions of age, gender, and TG trajectory class to gout risk prediction. **(E)** 5-year gout risk stratification pie chart based on TG trajectory patterns showing cohort distribution across different risk levels (low, medium, high, very high risk).

Latent class trajectory modeling based on longitudinal follow-up data successfully identified three distinct triglyceride (TG) dynamic evolution patterns (**Figure 4A**): stable high-level type (44.4%), improvement type (46.4%), and progressive elevation type (9.3%) (**Figure 4B**). Multivariable Cox proportional hazards regression analysis (**Figure 4C**) showed that using the improvement type as reference, patients with stable high-level type had significantly elevated gout incidence risk (HR = 1.92, 95%CI: 1.49-2.47, P<0.001). Variable importance analysis (**Figure 4D**) further confirmed that TG trajectory class was the third largest risk predictor after gender and age (relative contribution 35.5%). The 5-year risk stratification model constructed based on these findings (**Figure 4E**) showed that the model could effectively identify 23.9% of the cohort as medium-high risk population (predicted risk >10%), achieving precise early risk warning. Detailed Cox regression analysis results are shown in **Table 5**.

**Table 5:** Multivariable Cox proportional hazards model analysis results based on dynamic triglyceride trajectories.

Predictor	HR	95% CI	P-value
TG trajectory (Ref: Improvement type)			
Trajectory 2 (*vs*. Class 1)	0.91	0.79-1.06	0.227
Trajectory 3 (*vs*. Class 1)	1.92	1.49-2.47	<0.001
Age	1.01	1.00-1.01	<0.001
Gender (Male *vs*. Female)	2.01	1.71-2.37	<0.001
Hypertension (Yes *vs*. No)	0.80	0.69-0.91	<0.001
Diabetes (Yes *vs*. No)	1.02	0.86-1.21	0.834
Hyperlipidemia (Yes *vs*. No)	1.15	0.97-1.36	0.105

HR, hazard ratio; CI, confidence interval; TG, triglycerides.

### 3.7 Validation of causal association and genetic correlation between metabolic syndrome and gout

**Figure 5:**
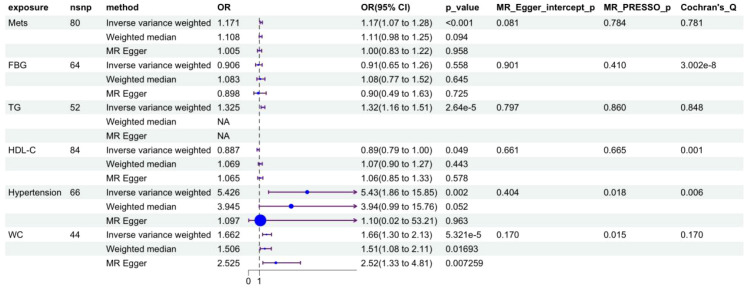
Forest plot of causal effects of metabolic syndrome and its components on gout risk. MetS, metabolic syndrome; WC, waist circumference; FBG, fasting blood glucose; HDL-C, high-density lipoprotein cholesterol; TG, triglyceride.

Two-sample Mendelian randomization (MR) analysis first established the causal effect between metabolic syndrome and gout (**Figure 5**): genetically predicted metabolic syndrome significantly increased gout incidence risk (OR = 1.171, 95%CI: 1.078-1.271, P<0.001). Among metabolic syndrome components, hypertension showed the strongest positive pathogenic effect (OR = 5.426, 95%CI: 1.854-15.883, P = 0.002), followed by waist circumference (OR = 1.523, 95%CI: 1.195-1.941, P<0.001) and triglycerides (OR = 1.325, 95%CI: 1.177-1.491, P<0.001), while HDL-C showed a significant protective effect (OR = 0.887, 95%CI: 0.786-0.999, P = 0.049).

**Figure 6:**
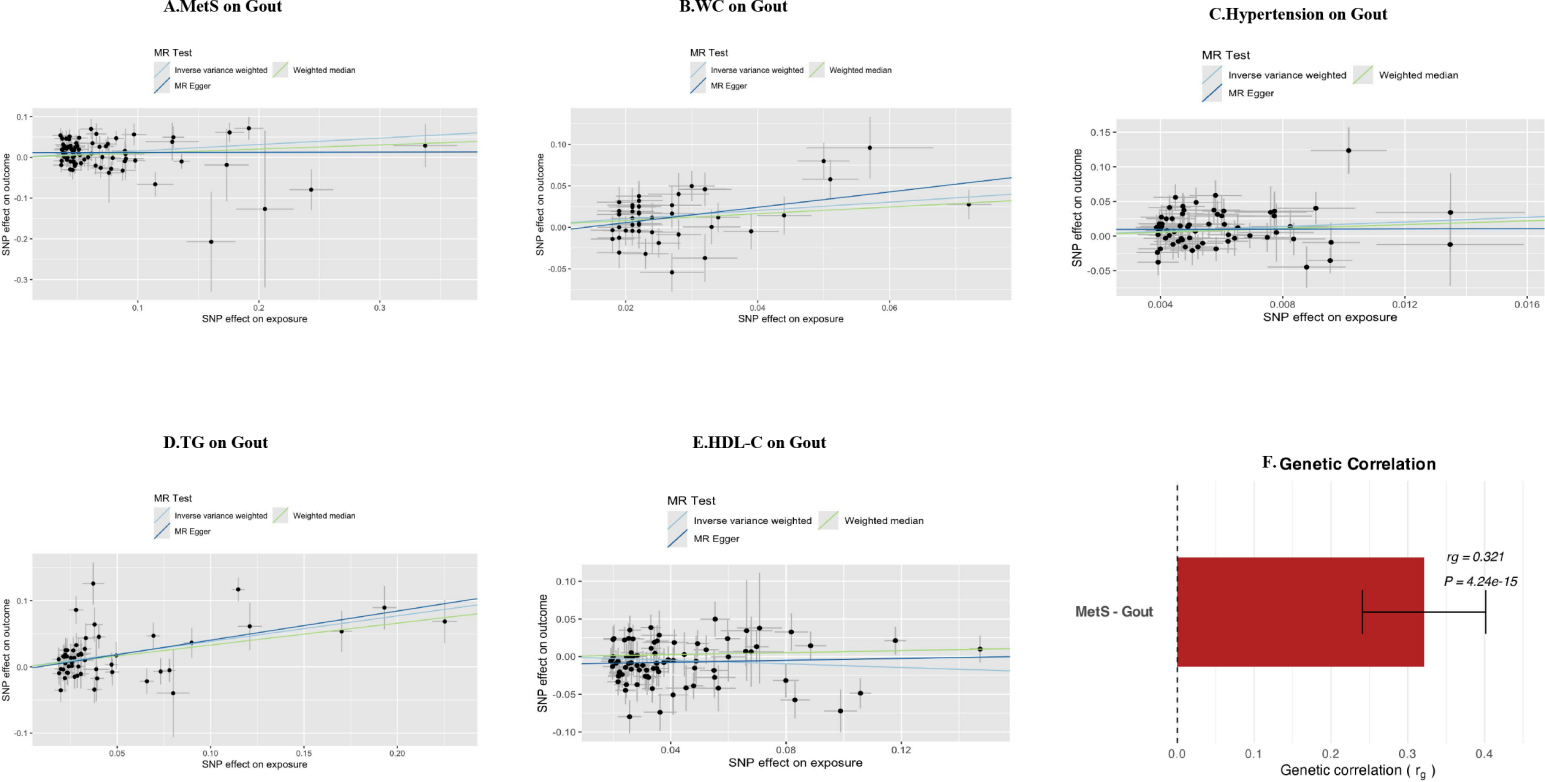
Visual validation of Mendelian randomization and genetic correlation analysis. **(A-E)** Scatter plots of causal associations between metabolic syndrome (MetS) and its components (waist circumference, hypertension, triglycerides, HDL-C) with gout. The x-axis represents SNP effect values on exposure factors (e.g., MetS), and the y-axis represents SNP effect values on outcome (gout). The slope of each line represents the causal effect estimate calculated by different MR methods (light blue: inverse variance weighted; dark blue: MR-Egger regression; light green: weighted median method). **(F)** Genetic correlation analysis between metabolic syndrome and gout based on LDSC. The height of the red bar represents the genetic correlation coefficient (rg = 0.321), and error bars represent standard error.

Linkage disequilibrium score regression (LDSC) analysis further confirmed at the genome-wide level that there was significant positive genetic correlation between metabolic syndrome and gout (rg=0.321, P = 4.24×10^-^¹^5^) (**Figure 6F**), providing solid genetic support for the above causal association. Scatter plots (**Figures 6A-E**) visually showed that the effect trends of each instrumental variable were highly consistent with inverse variance weighted (IVW) estimates. Leave-one-out sensitivity analysis (**Supplementary Figure 1**) confirmed that causal effect estimates were not driven by individual strong effect loci, demonstrating good robustness.

### 3.8 Common genetic architecture and molecular mechanisms of metabolic syndrome and gout

**Figure 7:**
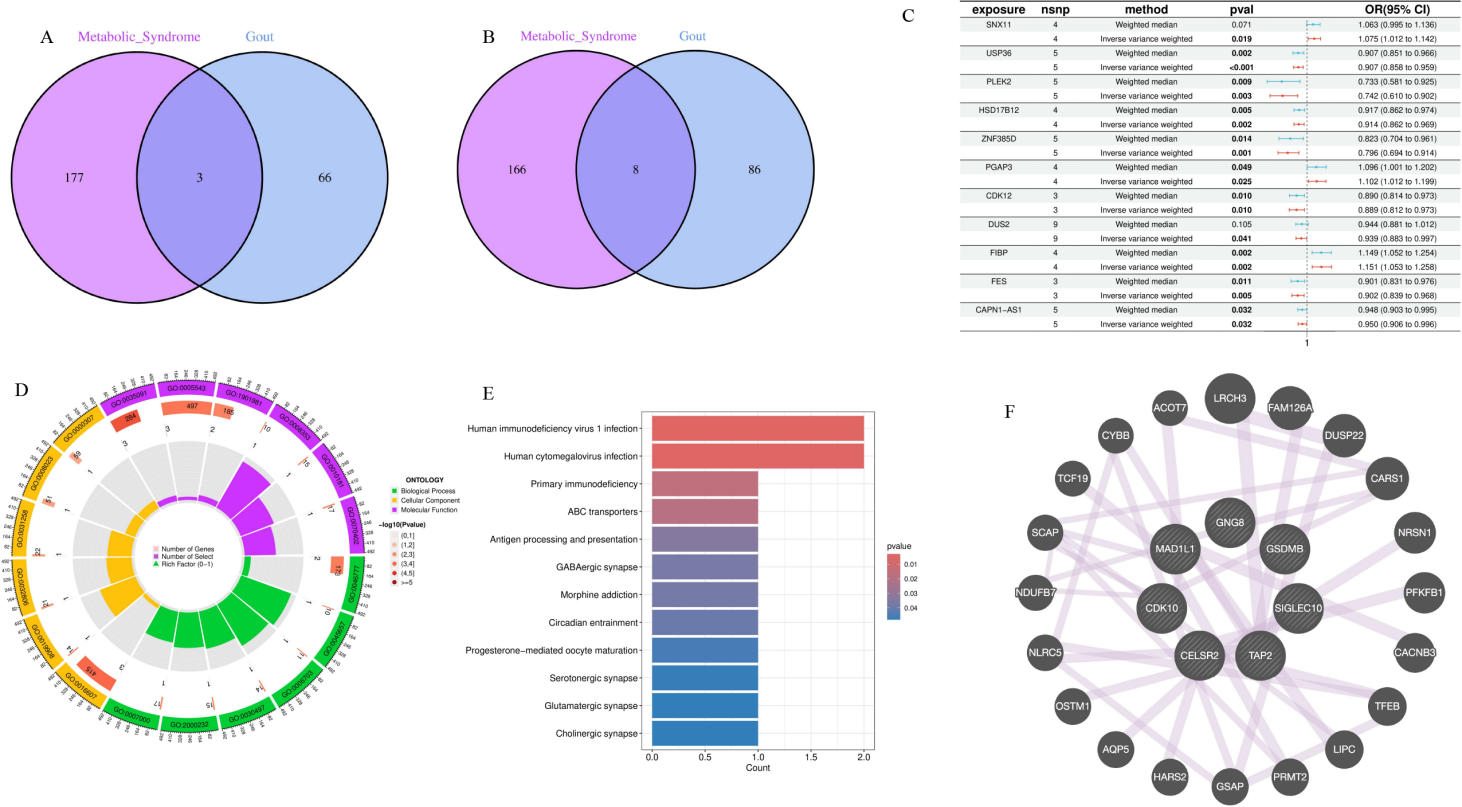
Identification of common pathogenic genes and mechanisms between metabolic syndrome and gout through Mendelian randomization and multi-omics integration analysis. **(A, B)** Venn diagrams showing common pathogenic genes of metabolic syndrome (purple) and gout (blue) identified by MR analysis. Figure **(A)** shows common risk genes (OR>1), and Figure **(B)** shows common protective genes (OR<1). **(C)** Forest plot summarizing causal effect estimates of identified common pathogenic genes on gout risk. Shows odds ratios (OR) and 95% confidence intervals calculated by inverse variance weighted and weighted median methods. OR>1 indicates risk genes (e.g., PGAP3, FIBP), OR<1 indicates protective genes (e.g., PLEK2, USP36). **(D)** Gene Ontology (GO) enrichment analysis circle plot of common pathogenic genes, showing significant enrichment terms for biological process (BP), cellular component (CC), and molecular function (MF). **(E)** Kyoto Encyclopedia of Genes and Genomes (KEGG) pathway enrichment analysis bar chart of common pathogenic genes. The x-axis represents gene count, and color gradient represents P-value significance. ABC transporters is one of the significantly enriched key pathways. **(F)** Protein-protein interaction (PPI) network of common pathogenic genes. Nodes represent proteins, and edges represent interaction relationships. Larger nodes indicate higher degree, suggesting they are hub genes in the network (e.g., MAD1L1, SIGLEC10).

LDSC analysis revealed highly significant positive genetic correlation between metabolic syndrome and gout at the genome-wide level (rg=0.321, P = 4.24×10^-^¹^5^), indicating that the two diseases share a large number of identical genetic risk variants, providing a genetic explanation for their comorbidity. To further identify specific molecular drivers, this study screened for dual pathogenic genes through gene-level MR analysis. Venn analysis revealed multiple overlapping pathogenic genes between metabolic syndrome and gout (**Figures 7A, B**). Causal effect analysis for gout risk (**Figure 7C**) showed that genes such as SNX11, PGAP3, and FIBP were identified as significant common risk factors (e.g., PGAP3: OR = 1.102, 95%CI: 1.012-1.199, P = 0.025; FIBP: OR = 1.151, P = 0.002). Conversely, genes such as PLEK2 (OR = 0.742, P = 0.003) and USP36 (OR = 0.907, P<0.001) showed significant common protective effects. Functional enrichment analysis further revealed potential pathological mechanisms. KEGG pathway analysis (**Figure 7E**) was significantly enriched for the ABC transporter pathway, which is highly related to uric acid transport mechanisms. Additionally, immune-related pathways such as primary immunodeficiency and antigen processing and presentation were also significantly enriched, suggesting that immune system dysfunction is a potential bridge for comorbidity of the two diseases. Protein-protein interaction (PPI) network analysis (**Figure 7F**) identified MAD1L1, SIGLEC10, and TAP2 as core hub genes in the network, among which SIGLEC10, as an immunosuppressive receptor, may play a key role in regulating the transition from metabolic inflammation to gout.

### 3.9 Transcriptomic characterization reveals immuno-metabolic cascade reactions linking metabolic syndrome and gout

**Figure 8:**
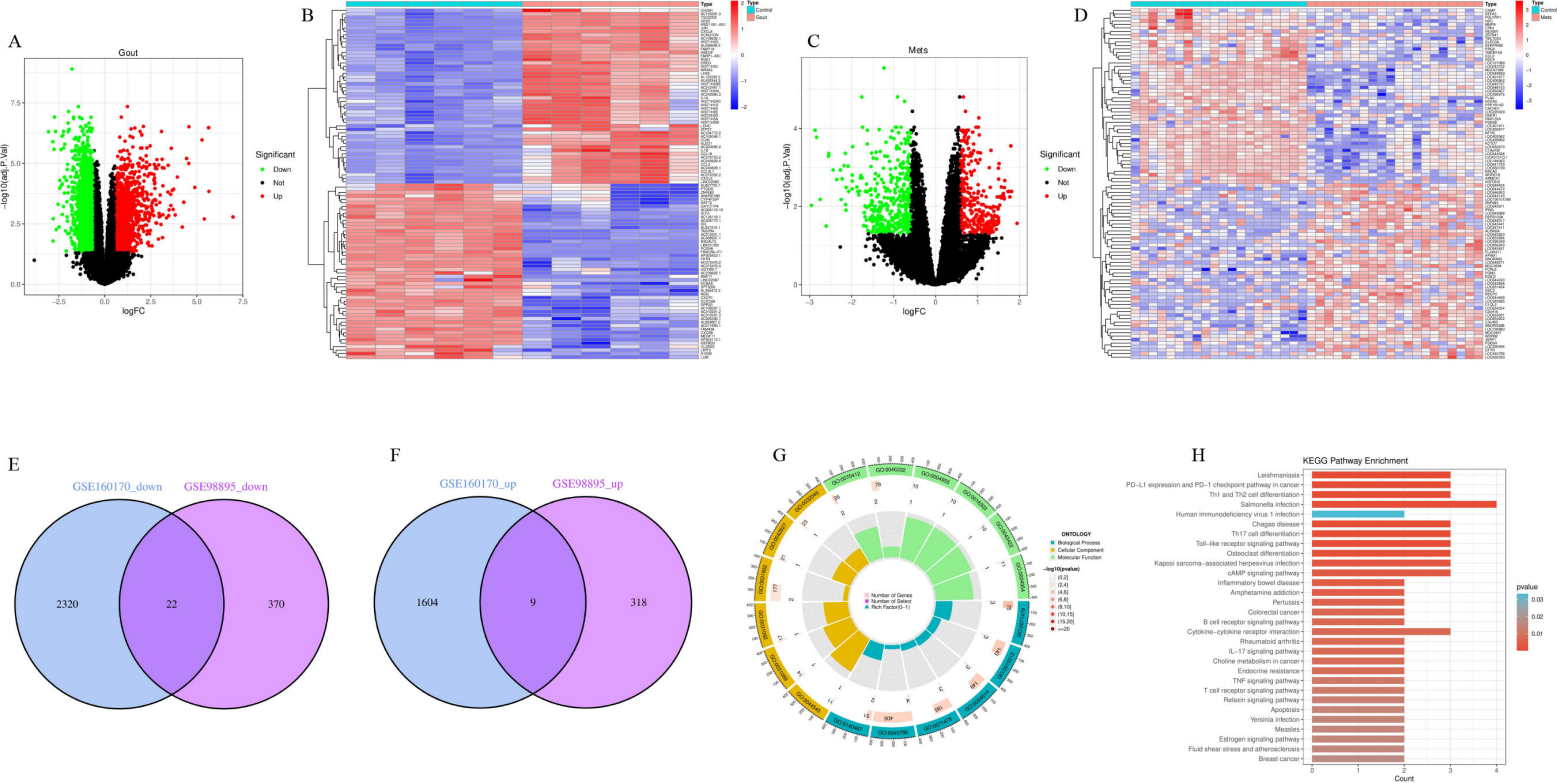
Transcriptomic analysis reveals downstream T cell activation cascade reactions complementary to upstream genetic susceptibility. **(A, B)** Differential expression analysis of gout dataset (GSE160170). Figure **(A)** shows volcano plot of significantly upregulated (red) and downregulated (green) genes in gout patients compared to healthy controls, Figure **(B)** shows heatmap of top differentially expressed genes. **(C, D)** Differential expression analysis of metabolic syndrome dataset (GSE98895). Figure **(C)** is volcano plot, Figure **(D)** is heatmap, visualizing transcriptomic changes in metabolic syndrome patients. **(E, F)** Venn diagrams showing intersection of differentially expressed genes between gout and metabolic syndrome. Figure **(E)** identifies 22 commonly downregulated genes, Figure **(F)** identifies 9 commonly upregulated genes, these genes represent core transcriptomic features linking the two diseases. **(G)** Gene Ontology (GO) enrichment analysis circle plot of common genes, categorizing significant enrichment terms into Biological Process, Cellular Component, and Molecular Function. **(H)** Scatter plot of Kyoto Encyclopedia of Genes and Genomes (KEGG) pathway enrichment. Results highlight downstream immune activation features echoing MR findings. Specifically, significant enrichment of ‘Th1 and Th2 cell differentiation’, ‘Th17 cell differentiation’, and ‘T cell receptor signaling pathway’ suggests that upstream antigen presentation defects may trigger downstream T cell overactivation. Additionally, the ‘Human immunodeficiency virus 1 infection’ pathway overlaps with genetic (MR) findings, confirming a common background of strong immune system activation.

To validate the pathological mechanisms suggested by genetic analysis at the transcriptomic level, this study analyzed transcriptomic data from peripheral blood mononuclear cells (PBMC) of gout (GSE160170) and metabolic syndrome (GSE98895) patients. Differential expression analysis showed that significant transcriptomic reprogramming occurred in both gout and metabolic syndrome patients compared to healthy controls (**Figures 8A-D**). Through Venn analysis, this study identified a group of core genes with co-expression changes in both disease states, including 9 commonly upregulated genes and 22 commonly downregulated genes (**Figures 8E, F**), representing the core transcriptomic features of comorbidity. Functional enrichment analysis revealed significant upstream-downstream logical associations between transcriptomic pathological features and the aforementioned MR findings. KEGG pathway analysis (**Figure 8H**) showed that human immunodeficiency virus type 1 infection was one of the significantly enriched pathways at the transcriptomic level, highly overlapping with genetically susceptible pathways identified by MR analysis, suggesting that both diseases are in states of strong immune defense and systemic overactivation during active phases. More importantly, multi-omics integration analysis depicted a clear antigen presentation-immune response pathogenic axis: MR analysis confirmed that antigen processing and presentation (particularly TAP2-mediated pathways) is the upstream source of genetic susceptibility, while transcriptomic data further captured the downstream effects of this defect—significant enrichment of Th1 and Th2 cell differentiation, Th17 cell differentiation, and T cell receptor signaling pathways. These results indicate that upstream genetic antigen presentation abnormalities may drive downstream adaptive immune polarization imbalance, particularly excessive activation of the Th17 axis, which is highly consistent with the chronic inflammatory states observed in gout and metabolic syndrome. Additionally, transcriptomic data also identified the involvement of AP-1 transcription complex-related genes including JUN and FOS, suggesting their potential role as executors of inflammatory cascade reactions in converting immune signals into persistent tissue inflammation.

### 3.10 Identification of core hub genes and construction of upstream regulatory networks

**Figure 9:**
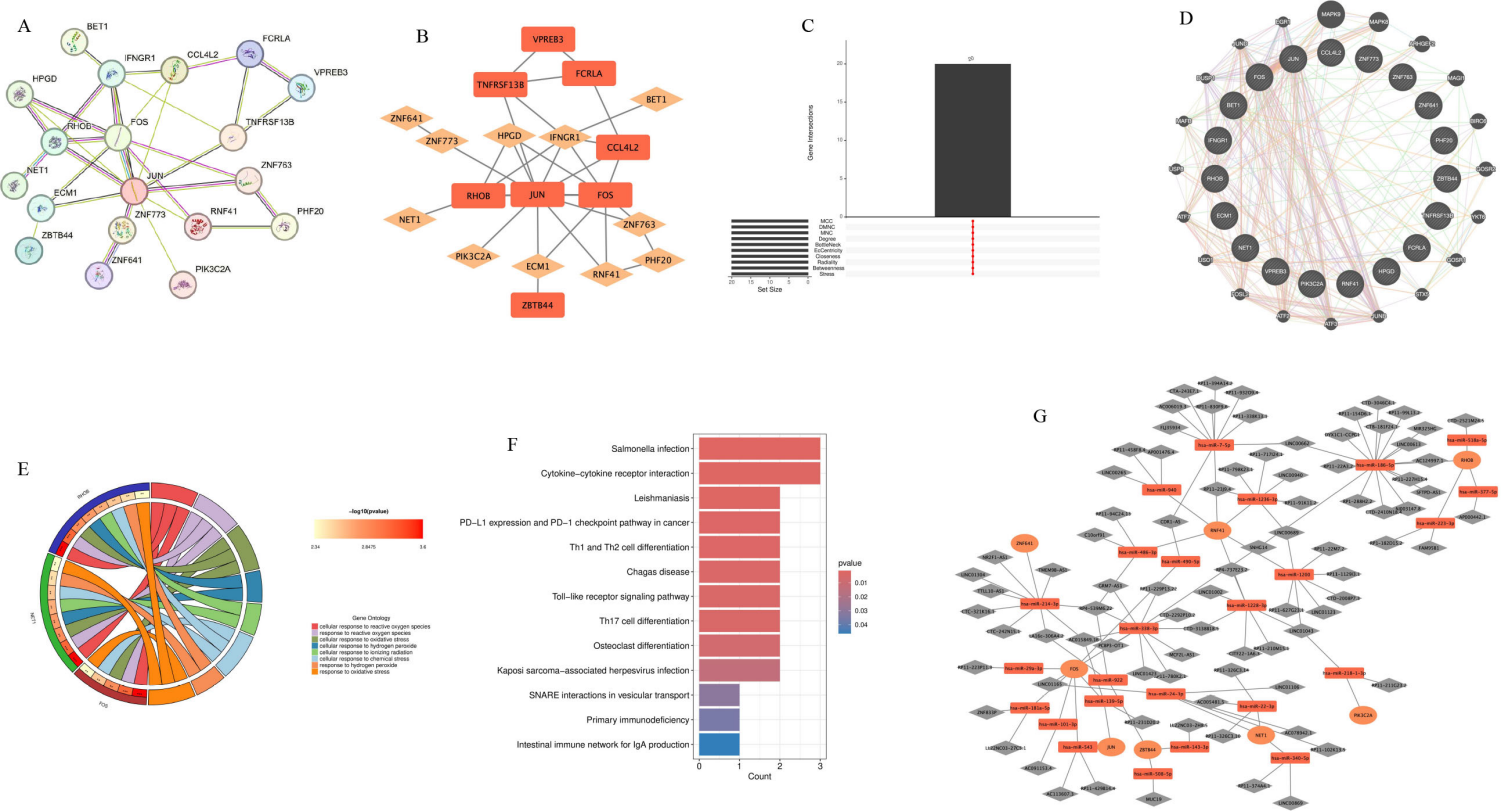
Identification of core hub genes and construction of multi-dimensional regulatory networks. **(A)** Global protein-protein interaction (PPI) network of common differentially expressed genes. **(B)** Visualization of core hub gene subnetwork, with node color depth representing topological importance. **(C)** UpSet plot showing hub gene screening strategy, with intersection representing robust genes simultaneously identified by 10 different topological algorithms. **(D)** GeneMANIA network analysis of hub genes, showing tight internal connections between genes based on co-expression (purple), physical interaction (pink), and pathway (blue). **(E)** Gene Ontology (GO) enrichment analysis circle plot of hub genes. **(F)** KEGG pathway enrichment bar chart of hub genes, emphasizing their roles in inflammatory signaling cascades. **(G)** Upstream transcriptional regulatory network constructed based on TRRUST database, with central nodes (orange diamonds) representing key transcription factors regulating peripheral hub genes (gray circles).

To identify key driver molecules from the complex comorbidity gene set, this study first constructed a PPI network of shared differentially expressed genes between metabolic syndrome and gout using the STRING database, and visualized it with Cytoscape (**Figure 9A**). Given the inherent limitations of single algorithms, ten different topological analysis algorithms from the cytoHubba plugin (including MCC, MNC, Degree, etc.) were employed to comprehensively evaluate the network. Through UpSet plot intersection (**Figure 9C**), core hub genes exhibiting high topological importance across all algorithms were ultimately identified. Visualization results showed (**Figure 9B**) that these hub genes (such as JUN, FOS, IFNGR1, etc.) occupy highly connected central positions within the network. GeneMANIA analysis (**Figure 9D**) further confirmed that these genes possess high internal connectivity at multiple levels including co-expression, physical interaction, and co-localization, constituting a tightly coordinated functional module. To validate the cell-type-specific expression patterns of hub genes at the cellular level, this study further utilized the gout-related single-cell RNA sequencing dataset (GSE217561) from the GEO database for electronic validation. Dot plot analysis revealed that hub gene IFNGR1 and TAP2, the key antigen presentation molecule identified from the MR-PPI network, exhibited the highest expression levels in monocytes and dendritic cells (**Supplementary Figure S2A-D**), supporting their functional localization in antigen-presenting cells. Further comparison between gout patients and healthy controls showed that JUN, FOS, and IFNGR1 were significantly upregulated in monocytes and dendritic cells of gout patients (all p < 0.0001) (**Supplementary Figure S2E, F**). Notably, TAP2 expression showed no significant difference between the two groups, which is highly consistent with our MR analysis conclusions - suggesting that the pathogenic effect of TAP2 stems from protein functional defects caused by genetic variants rather than changes in transcriptional expression levels. Single-cell validation confirmed the myeloid cell specificity of core hub genes at the cellular level, providing independent cell-resolution evidence for the “antigen-presenting cell dysfunction” mechanistic hypothesis. Functional enrichment analysis further characterized the biological roles of hub genes: GO circle plot (**Figure 9E**) showed that they primarily mediate oxidative stress response and immune cell activation, while KEGG analysis (**Figure 9F**) localized their functions to classical inflammatory signaling pathways such as IL-17, TNF, and Toll-like receptor pathways. To identify upstream molecular switches driving the aberrant expression of these inflammatory hub genes, this study constructed a transcription factor-target gene regulatory network based on the TRRUST database (**Figure 9G**), successfully identifying regulatory clusters centered on key transcription factors. These transcription factors regulate the expression of downstream target genes such as JUN and FOS, initiating and amplifying inflammatory signals, thereby establishing a complete molecular regulatory hierarchy from upstream transcription factors to core hub genes to downstream inflammatory pathways.

## 4 Discussion

Moving beyond a parallel multi-omics presentation, this study constructs a stepwise, mutually reinforcing causal validation framework to systematically dissect the comorbidity mechanism of metabolic syndrome and gout. The logical evidence chain is as follows: First, real-world clinical data provided macroscopic phenotypic evidence, identifying hypertension and hypertriglyceridemia as the most significant comorbid features of gout. Second, MR analysis overcame the confounding bias of clinical data, providing strong genetic evidence suggesting that these metabolic factors may have a direct causal role of these metabolic factors on gout, while ruling out the direct pathogenicity of hyperglycemia. Finally, transcriptomics filled the “black box” between “genetic causality” and “clinical phenotype,” revealing that “antigen presentation defects driving Th17 immune imbalance” may represent a potential molecular bridge connecting metabolic disorders and gouty inflammation. The main findings include: (1) Clinical cohort study confirmed MetS as an independent risk factor for gout, with significant non-linear relationships and population heterogeneity; (2) MR analysis confirmed causal associations between gout and MetS, hypertension, and triglycerides at the genetic level; (3) LDSC analysis revealed significant genetic correlation between them; (4) Multi-omics integration analysis identified the molecular mechanism of comorbidity centered on “antigen presentation-immune response.”

Our real-world clinical study provided strong clinical evidence. In a large cohort containing 8,853 subjects, after PSM matching to control confounding factors, MetS remained an independent risk factor for gout (OR = 1.456, P<0.001). This finding is consistent with previous epidemiological studies. A nationwide cohort study by Eun et al. (11) based on 3.5 million young Korean men showed that MetS patients had a 2.5-fold increased risk of gout incidence. Notably, among MetS components, hypertension (OR = 2.984) and hyperlipidemia (OR = 2.719) showed the strongest associations, suggesting that blood pressure and lipid control should receive particular attention in gout risk management. RCS analysis further revealed significant non-linear dose-response relationships between multiple metabolic indicators and gout risk, with most indicators showing “J” or “U” shaped curves with clear risk thresholds. For example, LDL-C showed lowest risk around 2.7 mmol/L, a finding of important guiding significance for clinically determining intervention cutoff points. Subgroup analysis revealed significant population heterogeneity: the association between MetS and gout was much stronger in males than females (P-interaction=0.007), while hyperlipidemia had greater risk impact on younger populations (P-interaction=0.007). These findings emphasize the necessity of adopting gender- and age-stratified individualized strategies in gout prevention.

To overcome the inherent limitations of observational studies in causal inference, we performed two-sample MR analysis. Results showed that genetically predicted MetS (OR = 1.171, 95%CI: 1.069-1.283, P<0.001), hypertension (OR = 5.426, 95%CI: 1.857-15.853, P = 0.002), triglycerides (OR = 1.325, 95%CI: 1.162-1.512, P<0.001), and waist circumference (OR = 1.523, 95%CI:1.195-1.941, P<0.001) had significant causal associations with gout risk, while HDL-C showed a protective effect (OR = 0.887, 95%CI: 0.786-1.000, P = 0.049). Notably, fasting blood glucose showed no significant causal association with gout risk (OR = 0.906, P = 0.558). Intriguingly, we observed discrepancies between our observational and MR findings regarding fasting blood glucose and LDL-C. While observational data suggested a protective or inverted J-shaped association, MR analysis did not support a causal effect. This discordance likely reflects reverse causation or physiological confounding in the real-world setting. For instance, the apparent protective effect of high glucose levels may be explained by the uricosuric effect of glycosuria (the “Bellomo phenomenon”), where severe hyperglycemia promotes uric acid excretion through osmotic diuresis (20). Similarly, the inverse association with LDL-C in the clinical cohort may be confounded by the use of lipid-lowering medications in patients with metabolic comorbidities or the lipid-lowering effects of chronic systemic inflammation (21). These findings highlight the superiority of MR in filtering out environmental confounders to identify true therapeutic targets. Multiple sensitivity analyses (MR-Egger regression, weighted median method) all supported the robustness of main results, and MR-Egger intercept test and MR-PRESSO global test did not detect significant horizontal pleiotropy. It is noteworthy that the strong causal effect of hypertension shown in MR analysis (OR = 5.426) echoes the high association observed in our clinical study, suggesting that hypertension may play a central role in gout pathogenesis. Biologically, hypertension can reduce uric acid excretion through renal microvascular damage (8), while renin-angiotensin system activation can upregulate urate transporter expression (22). LDSC analysis further confirmed significant positive genetic correlation between MetS and gout (rg=0.321, P = 4.24×10^-^¹^5^), indicating that the two diseases share a large number of genetic risk variants at the genome-wide level, providing a genetic basis for comorbidity.

Gene-level MR analysis identified a series of common pathogenic genes that simultaneously affect MetS and gout. Among them, PGAP3 (OR = 1.102) and FIBP (OR = 1.151) were identified as common risk genes, while PLEK2 (OR = 0.742) and USP36 (OR = 0.907) showed common protective effects. Functional enrichment analysis revealed the biological significance of these genes: KEGG pathway analysis was significantly enriched for the “ABC transporter” pathway, which is highly related to uric acid transport mechanisms. ABCG2 is known to be a key transporter for uric acid excretion in kidneys and intestines, and its dysfunction can lead to elevated serum uric acid (23). Additionally, significant enrichment of immune-related pathways such as “primary immunodeficiency” and “antigen processing and presentation” suggests that immune system dysfunction is a potential bridge for comorbidity of the two diseases. PPI network analysis identified MAD1L1, SIGLEC10, and TAP2 as core hub genes, among which SIGLEC10, as an immunosuppressive receptor, may play a key role in regulating the transition from metabolic inflammation to gout (24).

**Figure 10:**
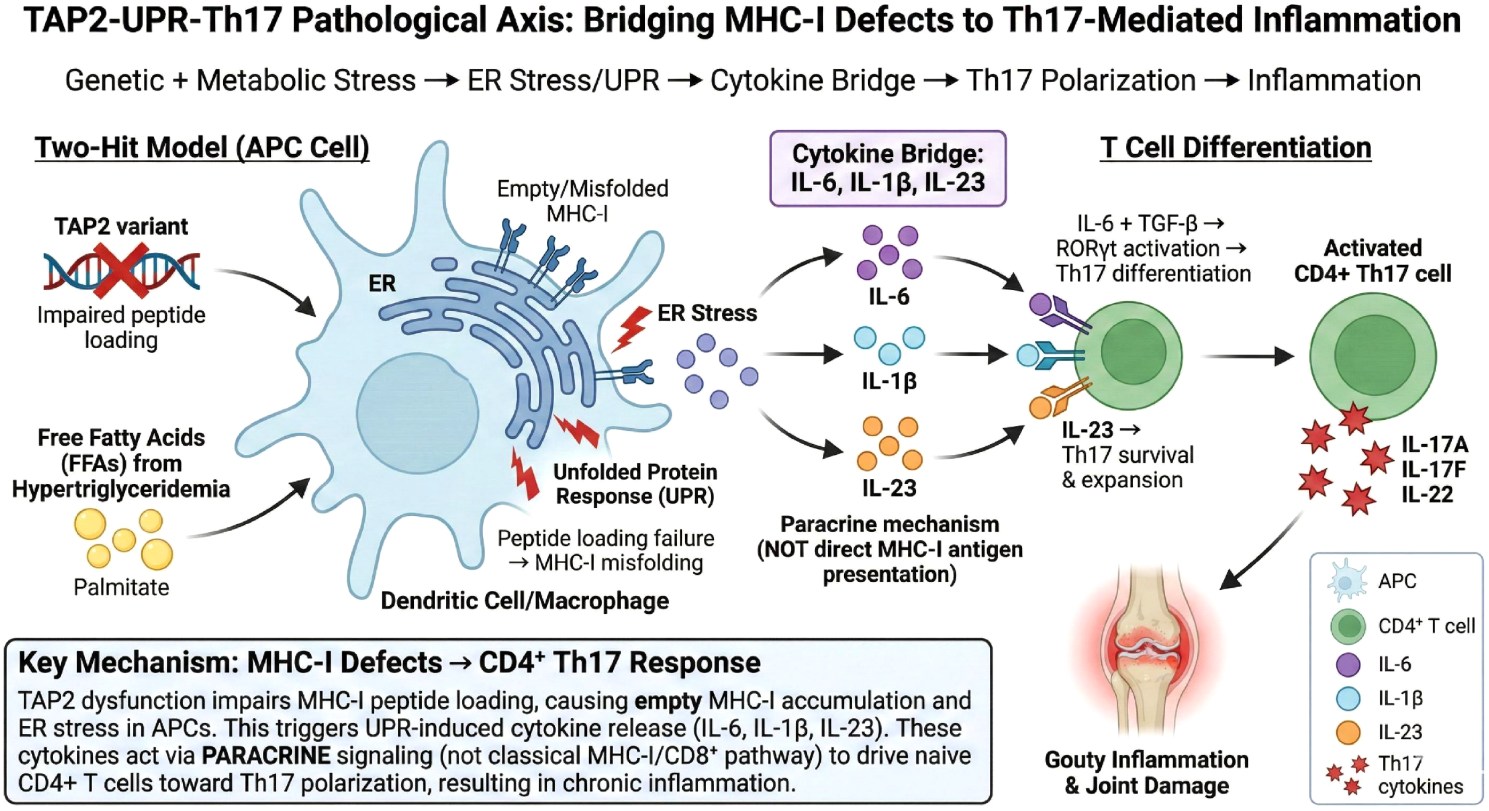
Proposed “Metabolic-Genetic-Immune” Comorbidity Mechanism Schematic (TAP2-UPR-Th17 Pathological Axis).This diagram illustrates the core pathological axis proposed in this study. The “Two-Hit Model” shows how metabolic syndrome-associated environmental stress (hypertriglyceridemia-derived free fatty acids) synergizes with genetic susceptibility (TAP2 variants). TAP2 dysfunction impairs antigen peptide transport, leading to empty MHC-I molecule accumulation in the endoplasmic reticulum and triggering the Unfolded Protein Response (UPR/ER Stress). Critically, this ER stress induces antigen-presenting cells to release a specific cytokine milieu—IL-6, IL-1β, and IL-23—which serve as the “Cytokine Bridge” connecting MHC-I pathway defects to downstream CD4+ T cell responses. Through this paracrine mechanism (distinct from classical MHC-I/CD8+ antigen presentation), IL-6 combined with TGF-β drives naive CD4+ T cell differentiation toward the Th17 lineage via RORγt activation, while IL-23 sustains Th17 survival and expansion. The resulting pathological Th17 response releases inflammatory mediators (IL-17A, IL-17F, IL-22), ultimately leading to the chronic inflammation and joint damage characteristic of gout.

To bridge the mechanistic gap between genetic causality and transcriptomic phenotypes, we propose a specific “Genetic-Stress-Immune” pathological axis. The TAP2 gene, identified by MR, is crucial for antigen peptide transport; its variants can lead to failure in MHC-I peptide loading. Evidence suggests that these conformationally unstable “empty” MHC molecules accumulate in the endoplasmic reticulum, triggering a persistent Unfolded Protein Response (UPR) (25). Notably, UPR activation is known to induce antigen-presenting cells to secrete cytokines such as IL-6 and IL-23, which create the critical microenvironment for Th17 cell differentiation (26). This mechanism may help explain the significant enrichment of the Th17 pathway observed in our transcriptomic data. Therefore, we tentatively hypothesize that the genetic background associated with metabolic syndrome (e.g., TAP2 variants) may primarily induce intracellular ER stress, which then drives the gout-characteristic Th17 adaptive immune response through cytokine crosstalk. However, this hypothesis requires further experimental validation. We acknowledge that in classical immunology, TAP2-mediated MHC-I molecules primarily engage CD8+ T cells through antigen presentation. However, we propose that the link between MHC-I pathway defects and Th17 polarization occurs through an indirect, paracrine mechanism rather than direct antigen presentation. Specifically, the accumulation of misfolded empty MHC-I molecules triggers ER stress and activates the UPR in antigen-presenting cells, particularly dendritic cells and monocytes (27). This chronic ER stress induces these cells to secrete a distinct cytokine milieu, notably IL-6, IL-1β, and IL-23 (28). These cytokines serve as the critical “bridge” converting intracellular MHC-I defects into extracellular immune polarization signals: IL-6 combined with TGF-β drives naive CD4+ T cell differentiation toward the Th17 lineage, while IL-23 sustains Th17 cell survival and expansion (29, 30). This paracrine cytokine-mediated pathway provides a mechanistic explanation for how genetic defects in the MHC-I loading machinery can ultimately result in CD4+ Th17-dominated inflammatory responses characteristic of gout. Furthermore, hypertriglyceridemia may synergize with TAP2 genetic variants to amplify ER stress in APCs. Elevated circulating triglycerides lead to increased cellular uptake of free fatty acids (FFAs), particularly saturated fatty acids such as palmitate. These FFAs can directly disrupt ER membrane lipid composition, activate protein kinase C (PKC) and c-Jun N-terminal kinase (JNK) signaling pathways, and induce reactive oxygen species (ROS) production, all of which converge to trigger or exacerbate ER stress (31, 32). Thus, we propose a “two-hit” model wherein the genetic background (TAP2 variants) provides the first hit by impairing peptide loading, while metabolic stress from hypertriglyceridemia delivers the second hit by independently inducing ER stress, together creating a synergistic amplification loop that drives pathological Th17 responses in metabolic syndrome-associated gout. Electronic validation based on single-cell database further supported this hypothesis. We utilized an independent gout single-cell RNA sequencing dataset (GSE217561) to validate the cell-type-specific expression patterns of core hub genes. Dot plot analysis showed that IFNGR1 and TAP2 exhibited the highest expression levels in monocytes and dendritic cells, supporting their functional localization in antigen-presenting cells. Further comparison between gout patients and healthy controls revealed that JUN and FOS were significantly downregulated, while IFNGR1 was significantly upregulated in monocytes of gout patients (all p < 0.0001). The downregulation of JUN and FOS may reflect negative feedback regulation or functional exhaustion of the AP-1 pathway under chronic inflammatory conditions, whereas the upregulation of IFNGR1 is consistent with sustained activation of the IFN-γ signaling pathway. Notably, TAP2 showed no significant difference in expression between the two groups (p = 0.39), a finding highly consistent with our MR analysis conclusions—suggesting that the pathogenic effect of TAP2 stems from protein functional defects caused by genetic variants rather than changes in expression levels. Single-cell validation confirmed the myeloid cell specificity of core hub genes at the cellular level, suggesting that monocytes/macrophages serve as the core origin of this pathological cascade (**Figure 10**).

Integrating multi-dimensional research findings, we propose a molecular mechanism framework for MetS and gout comorbidity: at the genetic susceptibility level, ABC transporter-related gene variants lead to uric acid transport dysfunction, while antigen presentation-related gene (such as TAP2) variants cause immune regulatory defects; at the metabolic disorder level, insulin resistance upregulates URAT1 expression to increase uric acid reabsorption (7), high triglycerides compete with uric acid for renal excretion pathways (33), and visceral fat secretes hypoxanthine as a substrate for uric acid synthesis (34); at the inflammatory amplification level, metabolic stress activates the NLRP3 inflammasome, free fatty acids enhance its response to MSU crystals (35), and Th17 cell overactivation maintains chronic inflammatory states. This mechanistic framework integrates the two main lines of uric acid metabolic abnormalities and immune inflammatory activation, providing a systematic theoretical explanation for understanding comorbidity.

This study has several methodological strengths. First, the multi-dimensional validation strategy (clinical cohort → genetic causality → molecular mechanism) constructed a complete evidence chain, enhancing the reliability of conclusions. Second, large-sample real-world data improved statistical power, and PSM method effectively controlled measured confounding factors. Third, MR analysis utilized the random allocation characteristic of genetic variants to maximize avoidance of reverse causation and unmeasured confounding influences. Fourth, multi-omics integration analysis validated findings at different biological levels (genome → transcriptome), revealing upstream-downstream associations of comorbidity mechanisms. Fifth, trajectory modeling captured the impact of dynamic evolution of metabolic indicators on disease risk, compensating for limitations of cross-sectional analysis. However, this study also has several limitations. First, the retrospective design of real-world study may have information bias and selection bias, and missing some important variables (such as lifestyle details) limits the completeness of confounding control. Second, GWAS data used in MR analysis mainly come from European ancestry populations, and the applicability of conclusions to other ethnicities remains to be verified. Third, although multiple sensitivity analyses were used, violation of MR assumptions (such as horizontal pleiotropy) may still affect results. Fourth, sample size for transcriptomic analysis was relatively limited, and being based on peripheral blood rather than joint synovial tissue may not fully reflect local pathological features. Fifth, this study is an association and causal inference study, and the proposed mechanistic framework still requires functional experimental verification. Future research should focus on: (1) validating MR findings in multi-ethnic populations; (2) conducting prospective cohort studies to clarify temporal relationships; (3) performing functional experiments to verify biological roles of key genes; (4) developing clinical risk prediction tools based on these findings.

In conclusion, this study systematically established the causal association between metabolic syndrome and gout through multi-dimensional evidence chains, revealing the molecular mechanism of comorbidity centered on “antigen presentation-immune response.” These findings emphasize the importance of monitoring and controlling metabolic factors in gout patient management, and provide new evidence-based foundation for developing risk stratification tools and targeted intervention strategies based on metabolic phenotypes.

## Data Availability

The real-world clinical cohort data used in this study are available from the corresponding author upon reasonable request. The transcriptomic datasets analyzed in this study are publicly available in the Gene Expression Omnibus (GEO) repository: the gout dataset (GSE160170) can be accessed at https://www.ncbi.nlm.nih.gov/geo/query/acc.cgi?acc=GSE160170, and the metabolic syndrome dataset (GSE98895) can be accessed at https://www.ncbi.nlm.nih.gov/geo/query/acc.cgi?acc=GSE98895. The single-cell RNA sequencing dataset used for electronic validation (GSE217561) can be accessed at https://www.ncbi.nlm.nih.gov/geo/query/acc.cgi?acc=GSE217561. The genome-wide association study (GWAS) summary statistics used for Mendelian randomization analyses were obtained from publicly available databases, including the IEU Open GWAS project (https://gwas.mrcieu.ac.uk), GWAS Catalog (https://www.ebi.ac.uk/gwas), and FinnGen database (https://finngen.gitbook.io/documentation). Detailed information on data sources for each exposure and outcome is provided in **Supplementary Table 1**.
