# Supplementary material for: Multi-dimensional evidence establishing the causal association between metabolic syndrome and gout and the molecular mechanisms of comorbidity
Source: Front Immunol. 2026 Feb 18;17:1769138. doi: 10.3389/fimmu.2026.1769138 (PMC12956786; doi:10.3389/fimmu.2026.1769138)
Supplement: Supplementary file 2 [file DataSheet1.docx]

Dear Editors,

We have uploaded all raw data, and images to the jianguoyun. Hope you can easily find these data following this link: https://www.jianguoyun.com/c/sd/1b06eb7/28904e66e4721650.

If you have any questions, please feel free to contact me.

Have a nice day

Best regards,

Wei Liu
