# Supplementary figures and images for: Multi-dimensional evidence establishing the causal association between metabolic syndrome and gout and the molecular mechanisms of comorbidity
Source: Front Immunol. 2026 Feb 18;17:1769138. doi: 10.3389/fimmu.2026.1769138 (PMC12956786; doi:10.3389/fimmu.2026.1769138)

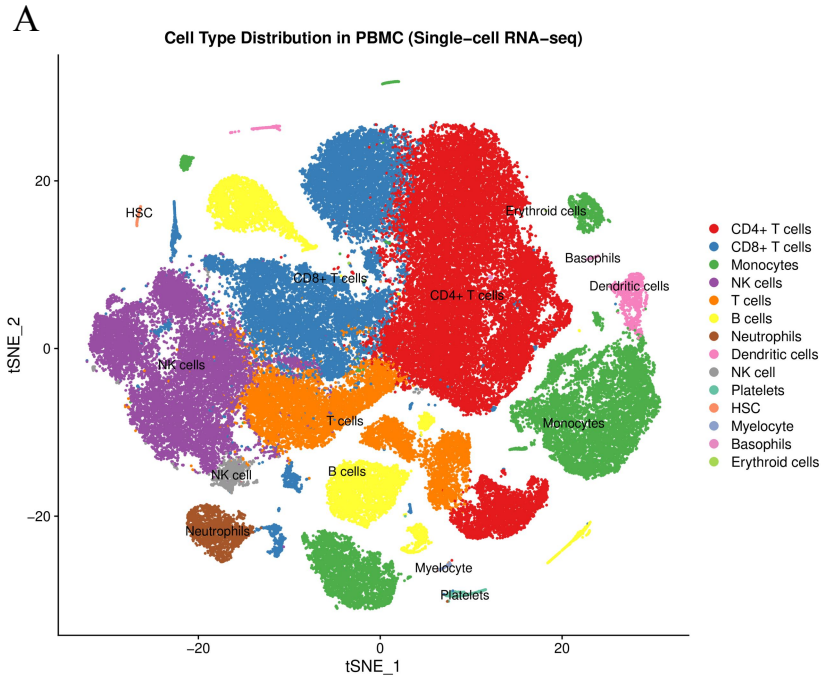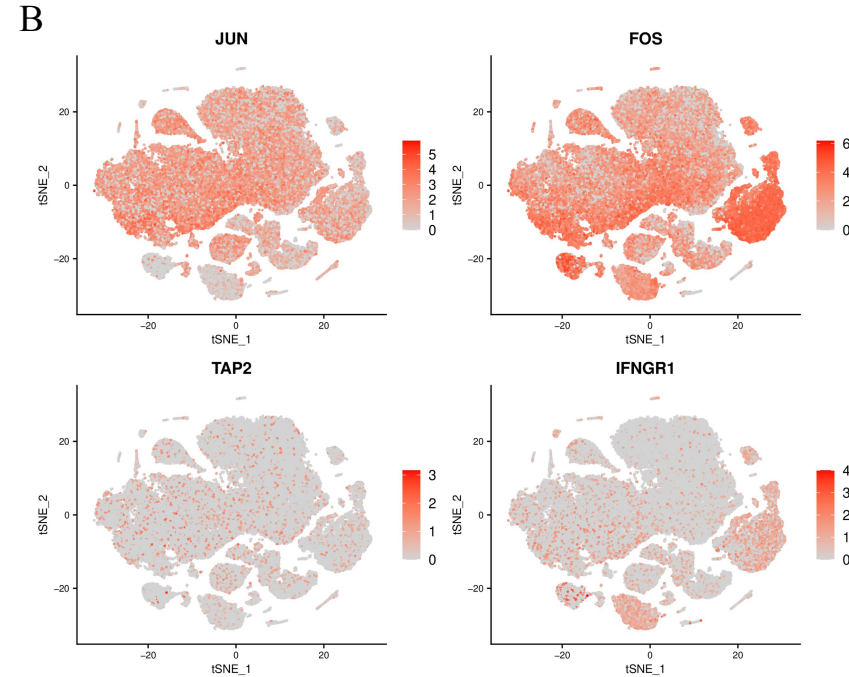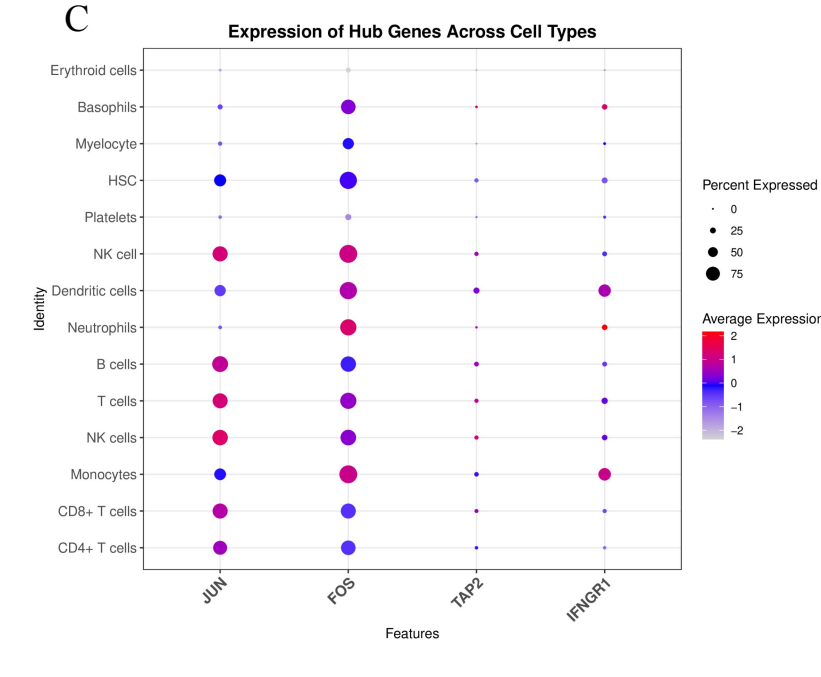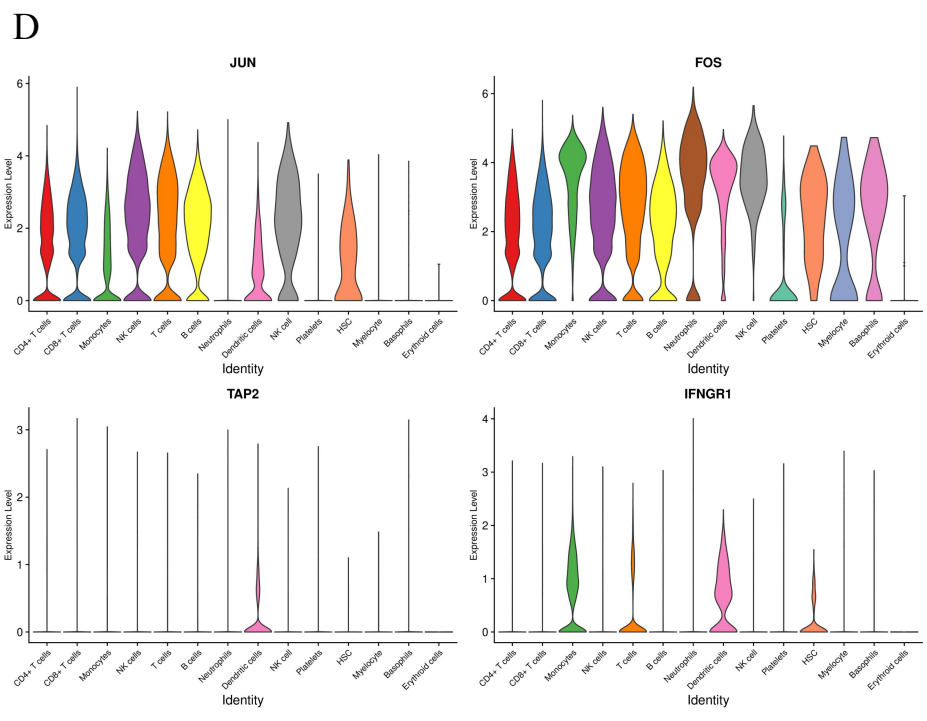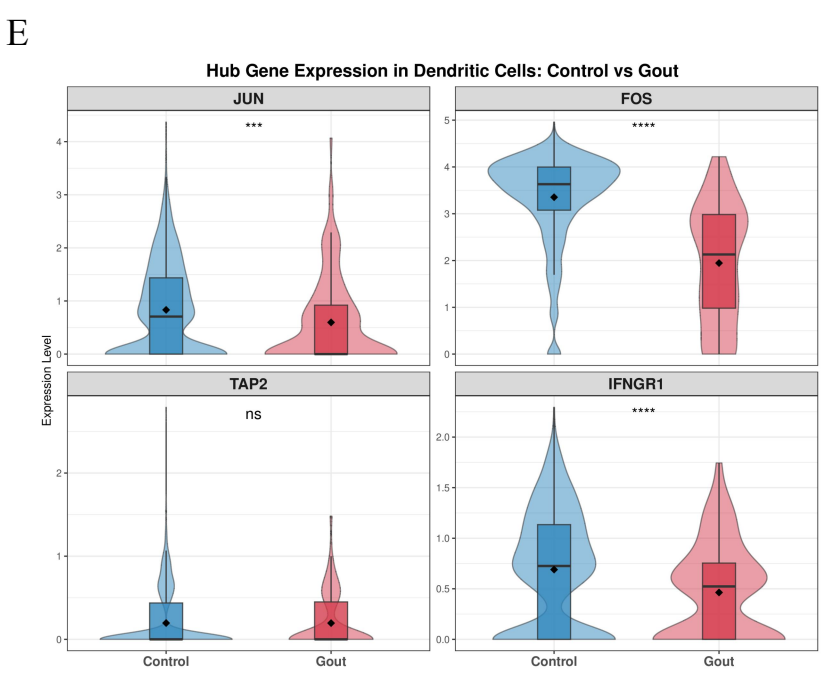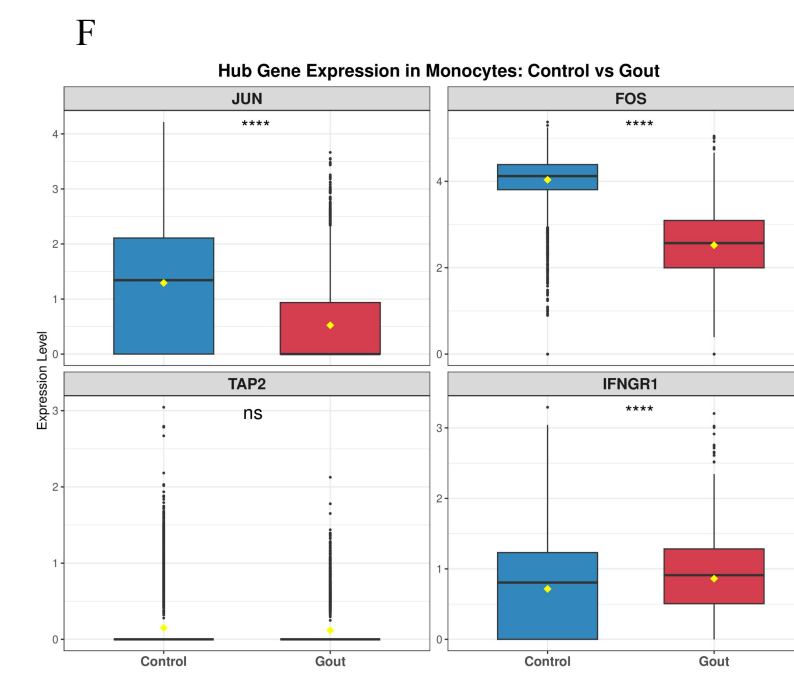

Supplement: Supplementary file 3 [file DataSheet2.pdf]
