# Supplementary material for: Multi-dimensional evidence establishing the causal association between metabolic syndrome and gout and the molecular mechanisms of comorbidity
Source: Front Immunol. 2026 Feb 18;17:1769138. doi: 10.3389/fimmu.2026.1769138 (PMC12956786; doi:10.3389/fimmu.2026.1769138)

MetS on Gout

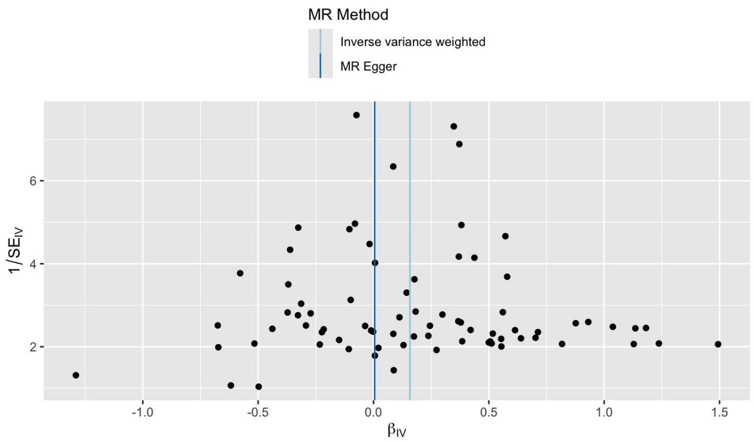

WC on Gout

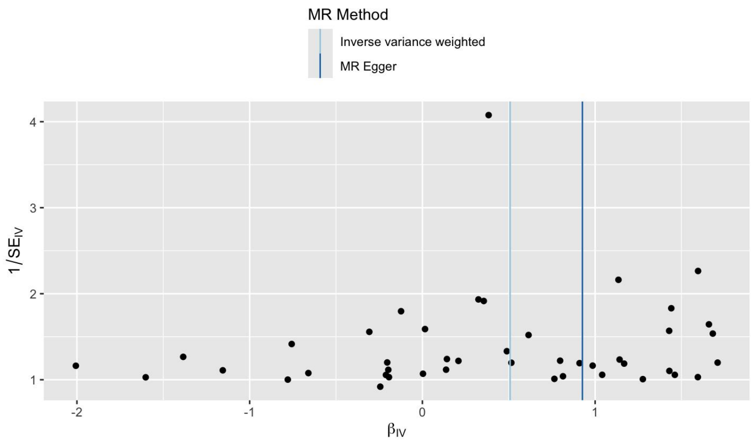

Hypertension on Gout

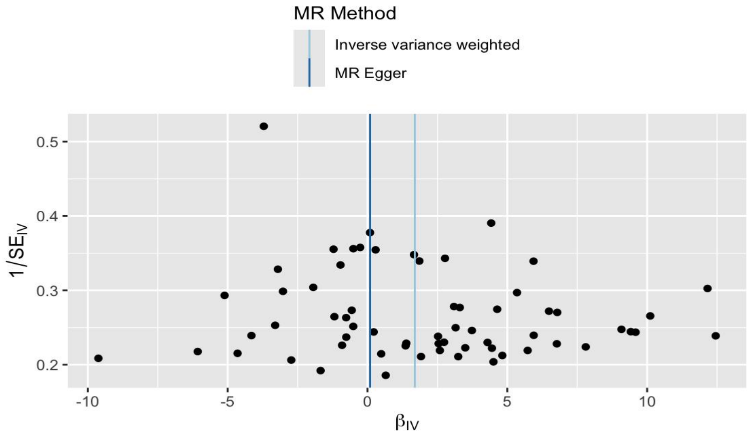

TG on Gout

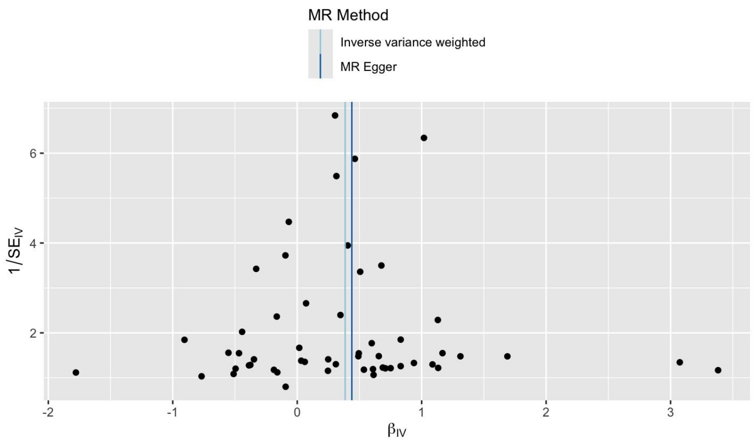

HDL-C on Gout

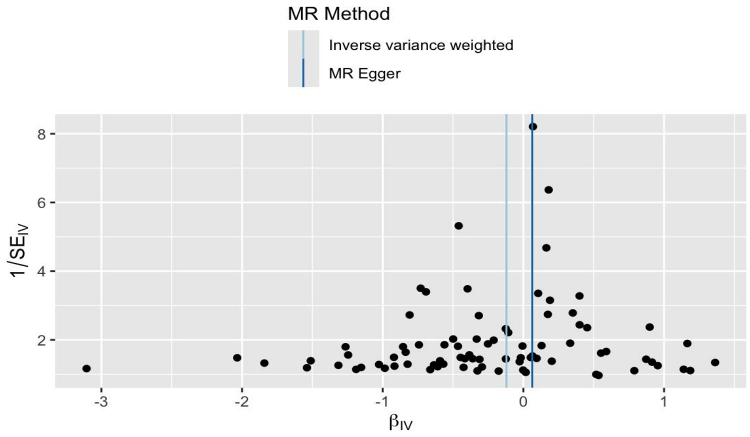

WC on Gout

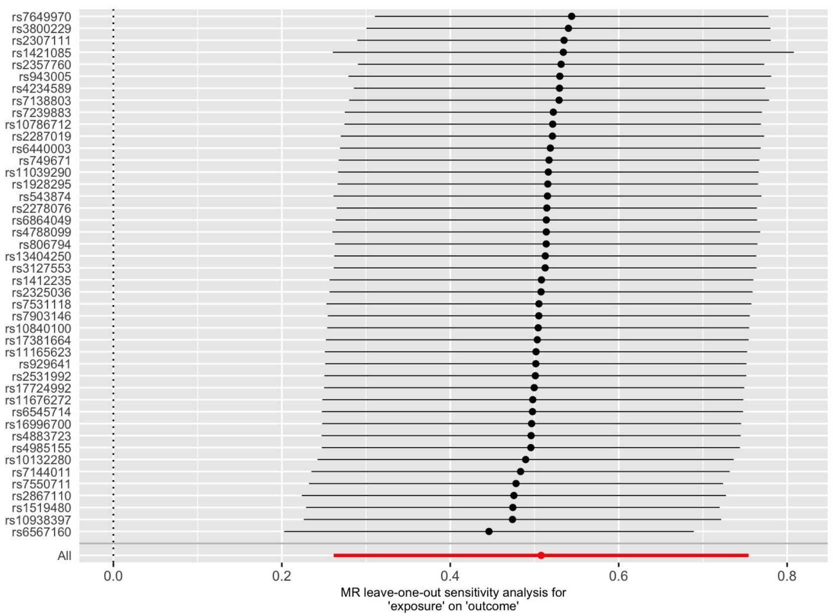

MetS on gout

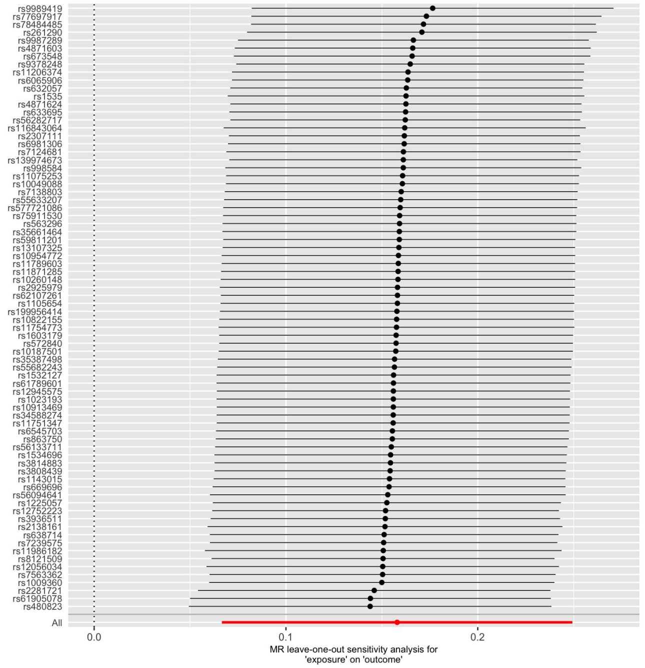

HDL-C on Gout

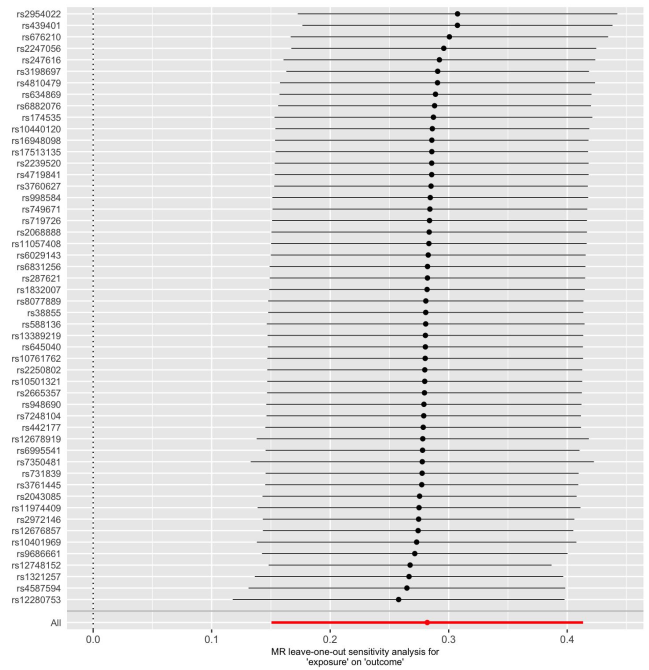

TG on Gout

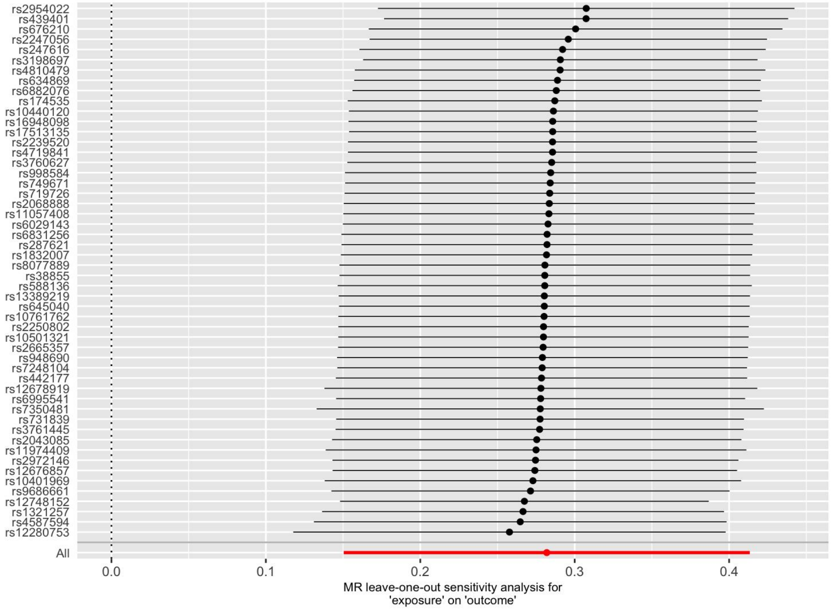

Hypertension on Gout

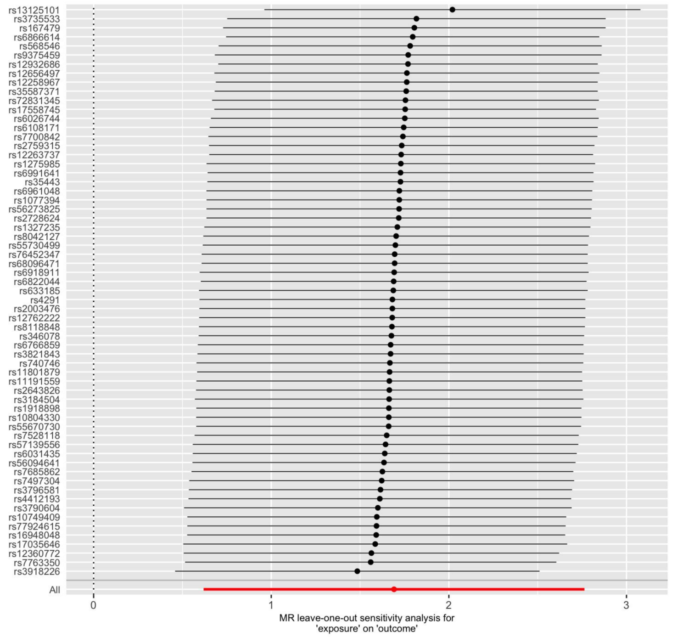

Supplement: Supplementary file 4 [file DataSheet3.pdf]
